# Supplementary material for: Detecting temporal and spatial malaria patterns from first antenatal care visits
Source: Nat Commun. 2023 Jul 6;14:4004. doi: 10.1038/s41467-023-39662-4 (PMC10326053; doi:10.1038/s41467-023-39662-4)
Supplement: Supplementary file 1 — Supplementary Information [file 41467_2023_39662_MOESM1_ESM.pdf]

Supplementary Information

Detecting temporal and spatial malaria patterns from first antenatal care visits.

Arnau Pujol, Nanna Brokhattingen, Glória Matambisso, Henriques Mbeve , Pau Cisteró, Anna Escoda, Sónia Maculuve, Boaventura Cuna, Cardoso Melembe, Nelo Ndimande, Humberto Munguambe, Júlia Montaña, Lídia Nhamússua, Wilson Simone, Kevin K.A. Tetteh, Chris Drakeley, Benoit Gamain, Chetan E. Chitnis, Virander Chauhan, Llorenç Quintó, Arlindo Chidimatembue, Helena Martí-Soler, Beatriz Galatas, Caterina Guinovart, Francisco Saúte, Pedro Aide, Eusébio Macete, Alfredo Mayor

Supplementary Methods

Quantitative suspension array assay

Coupling of antigens: Antigens (see **Supplementary Table 12**) were covalently coupled to beads following a modification of the Luminex® Corporation protocol[1]. Briefly, 2 ml of beads ($25x10^6$) were transferred into two 1.5 mL Eppendorf tubes and resuspended by sonication and vortexing. The supernatant was removed after precipitation of the beads by magnetic separation for 60 seconds. Beads were washed twice with 1.25 ml of distilled water and pellets were resuspended in 400 µl of activation buffer (0.1 M NaH2PO4, pH 6.2). Sulfo-NHS (N-hydroxysulfosuccinimide) and EDC (1-Ethyl- 3-[3-dimethylaminopropyl] carbodiimide hydrochloride; Pierce, Thermo Fisher Scientific Inc., Rockford, IL) dissolved in activation buffer were simultaneously added to reaction tubes at 5 mg/mL, and reaction tubes were incubated for 20 min at room temperature (RT) with gentle agitation and protected from light. Activated beads were washed twice with 1.25 µl of coupling buffer (MES 50 mM, 2-[Nmorpholino] ethanesulfonic acid monohydrate pH 5, Sigma-Aldrich). Recombinant antigens were coupled to the beads at 30 µg/ml, except peptides and GEXP18 which were coupled at 58 µg/ml and 1.7 mg/ml, respectively. Beads and antigens were vortexed, sonicated and then incubated overnight at 4°C in the dark, with shaking. Coupled beads were blocked with 1.25 ml 1% BSA in PBS for 30 minutes on a shaker at RT protected from light. Subsequently, beads were washed twice with a 1.25 ml assay buffer (1% BSA, 0.05% sodium azide in PBS filtrated) and resuspended in 1.25 ml of the same buffer for a final concentration of 10.000 beads/µl. Beads were quantified on a Guava PCA desktop cytometer (Guava, Hayward, CA), and stored in multiplex at 4°C in the dark.

DBS elution: 3 mm discs of blood dried onto filter paper containing approximately 2 µl of blood were obtained using an automated DBS punching machine (DBS Card Punch Machine, Analytical Sales & Services). 1 punched disc per sample was eluted in 50 µl Luminex Buffer in 96-well plates and placed onto a shaker at 4ºC overnight. Quality of elution was evaluated visually, and only samples with white filter paper and red elution were considered well eluted. Finally, the eluted samples were diluted 1:4 in Luminex Buffer for a final blood dilution of 1:100 with, and stored at 4ºC less than a week until the immunoassay was performed.

Bead-based immunoassay: Six controls were included in each plate. To monitor the DBS punching and elution process, mock DBS made from plasma mixed with pooled serum from 45 malaria-immune Mozambican pregnant women. A standard curve was prepared from serum pooled from 35 malaria-immune Mozambican pregnant women in a 3-fold 14-point serial dilution starting at 1:100 with Luminex Buffer (Phosphate Buffered Saline [PBS], 1% BSA, 0.05% azide, pH 7.4). Three dilutions of the standard curve (1:500, 1:5,000 and 1:50,000) were furthermore included to monitor variations between plates. As reference control, National Institute for Biological Standards and Controls (NIBSC) serum against *P. falciparum* (First WHO Reference Reagent for

Pf anti-malaria human serum, NIBSC 10/198) was included at 1:100 dilution in dH_2O. Furthermore, negative control samples collected from malaria-naïve individuals and blank wells were included in all plates. To measure the unspecific binding of antibodies in the sample to BSA used to block the beads, beads coupled to BSA were included in the bead multiplex.

50 µl of samples and controls were transferred to Luminex plates. Stock coupled beads were vortexed and diluted to 1,000 beads/well in Luminex Buffer, followed by 1 min bath sonication. 50 µl bead solution was loaded into each well and gently mixed by slow vortexing. Plates were incubated overnight at 4 ºC and 600 rpm on a shaker. The next day, plates were brought back to RT by agitation at 600 rpm for 1 h, before, plates were washed thrice in the wash buffer (PBS + 0.05% Tween-20 v/v (Sigma)). For the first wash, 100 µl wash buffer/well was added and the plates were placed onto a magnet for 2 minutes. For the second and third wash, 200 µl/well was added and the plates were placed onto the magnet for 1 minute. Wash buffer was discarded by flicking the plates. Detection antibody (Fc-specific anti-human IgG, Sigma-Aldrich) diluted to 1 µg/ml in Luminex Buffer was added at 100 µl/well, vortexed gently, and plates were incubated for 2 h at 600 rpm at RT. Plates were washed again using the procedure described above. Then streptavidin-phycoerythrin (Sigma-Aldrich) diluted 1:1000 in Luminex Buffer was added at 100 µl/well, and plates were incubated for 45 minutes at 600 rpm at RT. Plates were washed again, 100 µl/well of Luminex Buffer was added, and plates were kept at 4 ºC. The following day, plates were read on Luminex® 100/200™ instruments.

Acquisition of Luminex raw data was obtained using XPonent software. Protocols were created with the following parameters: MagPlex beads, maximum time per well: 60 seconds, High Photomultiplier Tube acquisition and Doublet Discriminator Gating from 5000 to 25000 nm. Measurements were discarded if fewer than 20 counts per recombinant antigen and 10 per peptide were obtained. The percentage of covariance between plates was calculated from the hyperimmune serum pool and considered acceptable if below 30%. A flowchart of the samples is found in **Supplementary Fig. 4**.

Data analysis

Estimation and comparison of positivity rates: Positivity rates of RDT for *Plasmodium falciparum* ($Pf\mathrm{PR}_{RDT}$) or qPCR positivity rates for *Plasmodium falciparum* ($Pf\mathrm{PR}_{qPCR}$) in the different populations of pregnant women were obtained as the mean positivity of RDT or qPCR results over the population. For the data from the cross-sectional surveys, a weighted mean was applied in the population using the representativity weight assigned to each individual to compensate for the oversampling of children from the study design.

In order to compare the positivity rates (PR) between pregnant women at ANC and children from the cross-sectional surveys, the PR from pregnant women was estimated as the mean positivity of all RDT or qPCR results between 45 days before and 45 days after the average date of each cross-sectional study (May 2017-2019), in order to obtain a sample size of the same order of magnitude for both populations. Moving this temporal window in time by 1-2 months around the cross-sectional dates had no significant impact on the results and conclusions of the study.

The relationship between $Pf\mathrm{PR}_{RDT}$ or $Pf\mathrm{PR}_{qPCR}$ of pregnant women and cross-sectional surveys was quantified with a linear regression between these measurements in the three areas of Magude Sede, Manhiça Sede and Ilha Josina and the three time periods of the cross-sectional surveys (May of 2017, 2018 and 2019). This made a total of nine estimations for each population, with the exception of qPCR data from Manhiça from the cross-sectional surveys, since it was not available for the study. The correlation between the positivity rates of both populations was quantified with the Pearson correlation coefficient (CC) between their estimations:

$$\rho_{X,Y} = \frac{\sum_{i=1}^{n}\left(x_i - \bar{x}\right)\left(y_i - \bar{y}\right)}{\sigma_x \sigma_y}, \quad (1)$$

where n is the total number of data points, x_i and y_i are the PR of pregnant women and cross-sectional surveys, $\bar{x}$ and $\bar{y}$ their mean, and $\sigma_{x,y}$ the standard deviation of the data sets. When we focused on low burden levels, in order to address the proportionally large error bars due to the low prevalences we have quantified the consistency between the positivity rates from the χ^2 statistics.

The consistency of the positivity rates between the populations was obtained from the χ^2 statistics:

$$\chi^2 = \frac{1}{n}\sum_{i=0}^{n} \frac{\left(PR_{ANC,i} - PR_{2-10,i}\right)^2}{\left(\sigma_{ANC,i}^{\ 2} + \sigma_{2-10,i}^{\ 2}\right)}, \quad (2)$$

where n is the number of measurements, $PR_{ANC,i}$ and $PR_{2-10,i}$ are the ith measurements of the positivity rate estimated from pregnant women and from children 2-10 years old respectively, and $\sigma_{ANC,i}$ and $\sigma_{2-10,i}$ are their respective error bars.

When restricting the χ^2 analysis to Magude and Manhiça (low transmission), given the degrees of freedom of the χ^2 statistics in this case, values below $\chi^2 < 2.2$ would correspond to a p-value 0.05 on a null-hypothesis statistical test, and values below $\chi^2 < 1.46$ would correspond to p-values 0.2, indicating a very good agreement between the two data sources.

Temporal analysis: In order to compare the temporal changes in transmission (or burden level) between data from clinical cases and from ANC, a temporal window of 6 months was defined for the temporal binning of the data to compare the temporal changes in transmission between clinical and ANC data. This binning was chosen in order to have enough time granularity to identify the temporal changes of transmission and obtain a stable estimation of their time lag but at the same time avoiding the ANC data to be too noisy (binning at smaller time scales would increase the error bars of the data due to the smaller sample size and the variations would have smaller statistical significance). This binning choice also included the rainy season inside the same bin in order to keep the sensitivity to seasonality. Then, *Pf*PR_{qPCR} (or *Pf*PR_{RDT}) from pregnant women was estimated as the average positivity of all the data inside the bin. For the clinical cases, the mean weekly RDT positive cases were obtained from all the weeks inside each temporal bin.

The linear correlation between the measurements of two different data sources was quantified using Pearson CCs. To quantify the agreement between the temporal variations in the PR from pregnant women and in the weekly number of positive cases in health facilities, χ^2 statistics of these estimations were calculated with a renormalization constant factor in the mean weekly RDT cases from clinical cases (the interest was to compare temporal changes, not in the absolute amplitudes). This constant factor was constrained by minimising the χ^2 statistics between the two estimations:

$$\chi^2 = \frac{1}{n}\sum_{i=0}^{n} \frac{\left(PR_{ANC,i} - kN_{CC,i}\right)^2}{\left(\sigma_{ANC,i}^{\ 2} + \sigma_{CC,i}^{\ 2}\right)}, \quad (3)$$

where n is the number of time bins, $PR_{ANC,i}$ is the positivity rate estimated from pregnant women in the ith time bin, $\sigma_{ANC,i}$ its error bar, $N_{CC,i}$ and $\sigma_{CC,i}$ are the mean number of weekly positive clinical cases and the error bar for the same bin, and k is the normalisation constant factor to constrain. k was considered a free parameter to optimise due to the different nature of the two temporal evolutions (one being a fraction between 0 and 1 and the other being an absolute positive number) and the fact that the interest was to compare the temporal changes and not the absolute numbers. Because of this, using an estimate of incidence instead of the number of clinical cases would give equivalent results since the denominator population was almost constant in this period and the normalising factor cancelled their difference in amplitude.

The statistical significance of the χ^2 statistics depends on the degrees of freedom (number of measurements) used in the different statistics. For each application of the χ^2 statistics in this study a reference threshold was defined that corresponds to a p-value 0.05 on a null-hypothesis statistical test, suggesting that χ^2 values below that threshold cannot be considered as inconsistent measurements. In the case of the temporal comparison analysis, 6 degrees of freedom (6 bins when using temporal windows of 6 months) corresponds to $\chi^2 \approx 2.17$.

Time lag between ANC data and clinical cases: In order to identify the time lag between two data sources, the Pearson CC between the two data sources was calculated by applying different time shifts between the ANC data (*Pf*PR_{RDT}, *Pf*PR_{qPCR} or seropositivity of a given antibody) and the data of clinical cases from passive surveillance. This was done systematically for the different populations of pregnant women and as a function of the time shift applied.

The time lag was chosen as the one with highest average Pearson CC between the three areas, since it was not possible to use all the areas together to estimate the Pearson CC since the clinical cases from each health facility had different unknown population denominators that could affect the correlation estimates when combining all the areas.

EpiFRIenDs method to detect malaria hotspots: The new software EpiFRIenDs, described in the next section, was used to detect malaria hotspots and serological clusters in this study. For the temporal analysis of data from ANC and from clinical data, EpiFRIenDs was run using a linking distance of 1km for the detection of hotspots and including cases in temporal windows of one month that were moved one day forward in each time step. A size threshold was applied to include only keep hotspots with at least three positive cases. The size threshold was applied in order to avoid false detections, and was defined by running 500 realisations of the datasets. In each realisation, all data was kept, with their geographical distribution but assigning infections from a probability defined by their overall prevalence. This allowed to study the detection of hotspots in the scenario that infections are not correlated with geographical location (i. e. there is no clustering of infections). From these runs, the average number of detected hotspots as a function of its size was obtained. Less than 0.1 hotspots were found on average with at least three positive cases, suggesting that the probability of having any least one false detection was lower than 10%. Hotspots (or clusters) from different time steps were linked if they were closer than 2km in space and closer than 30 days in time, considering them the same hotspot (or cluster) persisting in time. Since geolocation of clinical data from both positive and negative cases was only available in Manhiça district, the comparison has been limited to data from the Manhiça District Hospital and the Ilha Josina Health Centre.

Since EpiFRIenDs is a density-based clustering algorithm, its detectability depends on the density of the population, and different parameterisations of the algorithm would be required for dataset of

different densities. In order to compare the outcome of EpiFRIenDs from ANC data and from clinical data, which are datasets with very different sample sizes (3,616 geolocalized individuals (from which 351 were positive) from ANC data compared to 37,131 (from which 1,957 were positive) from clinical visits of children below 5 years of age in the same time period and in the two health facilities), the dataset from clinical data was randomly sub-sampled to obtain the same sample size of positive cases (that drive the first step of the identification of hotspots in EpiFRIenDs), corresponding to the 18% of the clinical data, so that the exact implementation of EpiFRIenDs could be used. Due to this downsampling of the clinical data, the result should not be interpreted as a comparison of the full potential of both data sources, but as a comparison of the highlighted high-risk areas and times of the different data sources. A hotspot from ANC data was considered to be matched by a hotspot from clinical data (or vice versa) if at least one member of an ANC hotspot was found closer than 2km to a member of a hotspot from clinical data. Five hundred different random sub-samples were done to run EpiFRIenDs on them in order to obtain statistical significance of the differences between the detected hotspots between ANC and clinical data. In each of the realisations, a different random selection of 18% of the clinical data was used, so that the sample size was kept without biasing the properties of the sub-samples.

When analysing the impact of HIV status and gravidity, the sample size of some of the populations used (such as HIV-positive women) limit the performance of EpiFRIenDs using the temporal windows of one month. For this reason, to assess the impact of the factors (HIV status, gravidity) a temporal window of one year was used, and EpiFRIenDs was run using the same linking distance of 1km for each of the three years of study and including the two districts of Magude and Manhiça.

Similarly, when comparing serological clusters of very different positivity levels, narrow temporal windows can affect differently to the sensitivity of EpiFRIenDs between the different antigens. For this reason, a temporal window of one year was used and the detection of hotspots and serological clusters from ANC data in each year was compared, using a linking distance of 1km, including all the data from each year and from the two districts of Magude and Manhiça. With these larger sample sizes, and following the same approach of running 500 realisations assigning random infections, the size threshold was increased to five positive cases per hotspot.

In this study, malaria hotspots were defined as areas with higher levels of infections than statistically expected in a stable period of time (using temporal windows of one month or one year). Serological clusters were defined from the term cluster used in the WHO malaria terminology that contemplates events that are not necessarily malaria cases[2]. In this way, the words hotspot and cluster in this study differentiate between *P. falciparum* infections and seropositivity.

EpiFRIenDs

Terminology

The World Health Organisation (WHO) proposed a terminology[11] for different spatial structures of interest for malaria epidemiology. This terminology includes the following terms:

- *Cluster:* Aggregation of relatively uncommon events or diseases in space and/or time in numbers that are considered greater than could be expected by chance.
- *Epidemic:* Occurrence of a number of malaria cases highly in excess of that expected in a given place and time.
- *Focus:* A defined circumscribed area situated in a currently or formerly malarious area that contains the epidemiological and ecological factors necessary for malaria transmission.
- *Outbreak:* A case or a greater number of cases of locally transmitted infection than would be expected at a particular time and place.

Although the definition of each term is different, their similarities imply that the same structure could be considered as any of these terms. Being the term focus the most inclusive one, the terms cluster, epidemic and outbreak require an excess of events or cases with respect to what would be expected. The term epidemic emphasises that this excess is high, and the term outbreak mentions that cases are locally transmitted, suggesting that the term is more appropriate for smaller geographical scales, but the terms lack a quantitative distinction between them. Although not included in the WHO terminology, the term hotspot is commonly used and defined as an area where malaria transmission is higher than average in a region. In this study, malaria hotspots were defined as areas with higher levels of infections than statistically expected in a stable period of time, and focus was used as the general term to refer to any spatial structure of interest.

Introduction

Identifying malaria foci in time and space allows for detection of malaria reservoirs in elimination settings and key areas for targeted interventions. The desired characteristics of the foci (e.g. hotspots or outbreaks) depend on the programmatic interest of the interventions and on the transmission settings in the area. For data-driven interventions, it is important to define such foci in a way that is at the same time relevant for the programmatic interest and consistent with the detection method. Despite the different definitions provided by the WHO malaria terminology, the connection between these definitions and their quantitative considerations remains undetermined, so that in practice different definitions are used for each particular setting and implementation.

The software SaTScan is the most commonly used approach for malaria hotspot detection and it identifies circular or elliptical areas with higher malaria prevalence than statistically expected. However, the method is limited by the irregular distribution of human populations, and the outcome is not necessarily connected with definitions of programmatic interest. This can limit the definition of hotspots to simpler approaches such as restricting analyses to administrative catchment areas, losing the potential of finer spatial granularity. Methods that account for population distribution while maintaining operational feasibility are needed to improve the effectiveness of targeted interventions.

Epidemiological Foci Relating Infections by Distance (EpiFRIenDs) is a newly developed method to detect foci that accounts for population density by linking positive cases of a disease (e.g. malaria) between them when they are closer than a given distance of programmatic relevance, identifying structures of any shapes and sizes. The method can identify persistent foci that also evolve in time. The parameters of the method are of easy physical interpretation in order to easily connect the programmatic interests with its particular implementation. The method is presented here, with a

comparison of EpiFRIenDs with SaTScan using mock and real data, showing a better capacity to adapt to irregular population distributions. The method is publicly available in R[12] and Python[13].

Method

EpiFRIenDs is inspired by the Friends-of-Friends algorithm, commonly used in the field of cosmology to detect clusters of galaxies or dark matter haloes[14], and the density-based spatial clustering of applications with noise (DBSCAN) algorithm[15], a generalisation of Friends-of-Friends. The main adaptation comes from the incorporation of control groups to better quantify the statistical significance and the positivity rates of the foci found. Briefly, EpiFRIenDs identifies foci by linking positive cases when they are closer than a given pre-defined linking distance and indirectly linking them to all the positive cases that are linked to their connections. This creates groups of positive cases that are connected through this linking distance. Then, this group is extended by including the negative cases that are closer than the linking distance to at least one of the positive cases from the group. With this, EpiFRIenDs outputs a catalogue of foci with information such as the mean geographical location, the number of positive and negative cases of the foci, the positivity rate of the foci and the p-value of the statistical significance of the detection.

Input data

The required input data is a catalogue of cases with their diagnostic test results (a binary variable) and their geographic location (it can be either in cartesian coordinates or in latitude and longitude). The date time of the cases can also be used for a temporal analysis of the foci in order to quantify the persistence of hotspots.

Input parameters

The linking distance *link_d* has to be specified as an input parameter. This distance is used to link cases when they are closer than this distance, and its choice can be based on the overall spatial density of cases (the average distances between them), potential biological factors (e.g. mosquito travel distances) and the programmatic interests (the spatial granularity or connectivity between cases). Optionally, a minimum number of neighbours *min_neighbours* can be defined to only link cases when there are at least *min_neighbours* cases within the radius of the linking distance.

For the temporal analysis of foci, a linking time and distance needs to be defined to link foci from different time steps when they are closer than this linking time and distance, assigning to them the same focus temporal identifier. Additionally, the initial and final dates and the temporal window of each time step can be set to define the time steps of the analysis.

Algorithm

The algorithm involves two steps:

1. Running the DBSCAN algorithm on positive cases: the predefined linking distance *link_d* is used to connect cases that are closer than this distance. The default implementation has a minimum number of neighbours *min_neighbours*=2, which corresponds to the Friends-of-Friends algorithm. After identifying all the neighbours (cases closer than *link_d*) of all cases, the cases are linked directly to all their neighbours (if at least *min_neighbours* neighbours are found), labelled as friends, and indirectly to all the cases that are linked to their friends (friends of friends). This creates clusters of positive cases that are close to each other and that we define here as foci.
2. Extending the foci with negative neighbours: each focus of positive cases is extended to include all negative cases that are friends (but not friends-of-friends), so closer than *link_d,* to at least one of the positive members of the focus. The final foci will consist of groups of positive and negative cases, with at least *min_neighbours* positive cases. The statistical significance of

each focus is determined from the null-testing hypothesis that the probability, for each already detected focus, of having at least its number of positive cases is just given by the prevalence of the whole data set.

There is also the option of applying a temporal analysis of the EdiFRIenDs method. In this case, the algorithm is applied to all the time periods available defined from the initial and final dates of the cases and a temporal window to include the cases in each iteration. Foci from different time frames will then be linked if they are closer than the predefined linking geographical distance and linking time. Linked foci are then considered as the same foci persisting in time. A focus temporal identifier (temporal ID) is assigned to the foci, assigning the same temporal ID to those foci that have been linked, and iterating over all time frames. This approach can produce merging events, assigning the same temporal ID to different foci from the same time step due to their merging in past or future times.

Output

The output is a foci catalogue will contain this information for each focus:

1. Focus ID
2. Mean geographical position of positive cases
3. Mean geographical position of all (positive and negative) cases
4. Positivity rate (Fraction of positive cases)
5. Number of positive cases
6. Number of negative cases
7. Total number of cases
8. Index identifier of all the focus members
9. P-value of the statistical significance of the detection

When a temporal analysis is done, the catalogue also provides:

1. Mean date of the time step
2. The temporal ID
3. Earliest date of the cases of its temporal ID
4. Latest date of the cases of its temporal ID
5. Lifetime (time interval) of the detection of the temporal ID

Analysis

SaTScan

SaTScan[16] was used in this analysis in order to compare the performance of EpiFRIenDs with this state-of-the-art approach in different situations. Being one of the most common approaches for hotspot detection in the literature, SaTScan can use different statistical models (typically Poisson or Bernoulli) to detect hotspots by identifying circular or elliptical areas where the positivity rate is higher than statistically expected[17,18]. These areas are found by analysing the areas centred in the data points and varying the size of the area up to a window size limit defined as the fraction of the total population covered by the area. The hotspots are finally defined from the non-overlapping areas with highest statistical significance.

Mock data and methods

The performance of EpiFRIenDs was tested and compared with SaTScan using simulated data with a prevalence of 20% reproducing three different scenarios visualised in **Supplementary Fig. 7**. The first one, where both positive and negative cases were randomly distributed in space (**a**). The second one, where four circular clusters of positive cases were simulated on top of a

background random distribution of negative cases (**b**). Finally, the positive cases were distributed in a sinusoidal shape (**c**) in order to show the performances when populations are distributed elongated and irregularly shaped. The two methods were applied using four different spatial scales in order to visualise the impact of their parameters on the detection of hotspots. For EpiFRIenDs, the linking distances used were 0.01, 0.02, 0.05 and 0.1. For SaTScan, the method used for the maximum window sizes including 2%, 5%, 10% and 30% of the total population.

Application to mock data

The foci identified in EpiFRIenDs and SaTScan for the first population (both positive and negative cases randomly distributed) are shown in **Supplementary Fig. 8** and **Supplementary Fig. 9** respectively. The coloured points show the positive cases that belong to a hotspot, and their colour indicates their p-value. Grey points show the locations of negative cases and positive cases that do not belong to any hotspot. Foci with $p<0.05$ were only found in the smallest scales of EpiFRIenDs (linking distances of 0.01 and 0.02) for very small hotspots (1 containing 4 cases and 7 containing 2 cases). This is because at these very small distances we find many isolated pairs of cases and, given the prevalence used in the simulation (20%), hotspots with 2 positive cases and 0 negative cases have a p-value of 0.04. In other words, 4% of random pairs of cases would be both positives by chance. These were considered false detections expected from the choice of the linking distance, and this analysis actually indicates that only hotspots with more than 2 cases should be included. In fact, this scenario is unlikely to be applied in real data, since clusters of only 2 cases in scenarios with prevalence of 20% would not be programmatically relevant. For the application of EpiFRIenDs to real data in our analysis, the fraction of expected false detections in each data was estimated by generating 500 different realisations of the data sets, assigning their infections randomly to the data points and running EpiFRIenDs on them. These outputs the average number of foci detected as a function of the size (number of cases), allowing to determine the minimum size required to avoid false detections.

The foci found with EpiFRIenDs and SaTScan for the second population (simulating four circular clusters) are shown in **Supplementary Fig. 10** and **Supplementary Fig. 11** respectively. In both methods, many small foci were found when small scales were used, and fewer when larger scales were used. In the largest scales (**Supplementary Fig. 10d**, **Supplementary Fig. 11d**) the three rightmost clusters were considered only one and only two foci were found in total. Intermediate scales (**Supplementary Fig. 10c**, **Supplementary Fig. 11c**) would be more appropriate to characterise these structures. Significant ($p<0.05$) and large (more than 10 cases) were found for linking distances larger than 0.02 (**Supplementary Fig. 10b**) and maximum window sizes of 5% of the population (**Supplementary Fig. 11b**). Finally, the results of the population with a sinusoidal distribution of positive cases are shown in **Supplementary Fig. 12** and **Supplementary Fig. 13** for EpiFRIenDs and SaTScan respectively. In this case, EpiFRIenDs identified significant ($p<0.05$) and large (more than 10 cases) foci from the shortest linking distances already, identifying the regions with densest positive cases (where the maxima and minimum of the sinusoidal curves were located). Larger linking distances grouped larger fractions of the structure, with the extreme of finding only 2 foci for a linking distance of 0.1 (**Supplementary Fig. 12d**). On the other hand, SaTScan only found two significant foci using maximum window sizes >= 5% of the population, limited to the very extremes of the first maximum and the minimum of the sinusoidal curve.

Application to real data

Supplementary Fig. 14a shows the spatial distribution of households from pregnant women attending first antenatal care (ANC) visits in Magude and Manhiça districts in Southern Mozambique during 2017 (*Plasmodium falciparum* (*Pf*) positive from quantitative polymerase chain reaction (qPCR) shown in red, *Pf*-qPCR negative shown in green). **Supplementary Fig. 14b**

shows in colours the positive cases belonging to hotspots detected by EpiFRIenDs using a linking scale of 1000m, with the colour representing the qPCR positivity rate of the hotspot. **Supplementary Fig. 14c-d** shows the detections of EpiFRIenDs and SaTScan using different scales for both methods. The map is zoomed around Ilha Josina because it is the only region where SaTScan detected any hotspot and hence it is the only area of interest for the comparison. The structures identified by SaTScan depended strongly on the maximum window size used, while the structures identified by EpiFRIenDs from using different linking distances mainly differed on the merging of such structures.

Discussion

This is the official presentation of the new method, EpiFRIenDs, to detect epidemiological foci of arbitrary shapes and sizes based on the Friends-of-Friends and DBSCAN algorithms. The method only requires the geographic location and diagnostic test result of the data set and outputs a catalogue of foci with information about the number of negative and positive cases, mean geographical location, positivity rates and statistical significance of the detection. Also, a temporal analysis can be done to quantify the stability and persistence of foci in time if dates are provided.

EpiFRIenDs and SaTScan were used to compare their performance on detecting foci in different simulated scenarios. EpiFRIenDs can output false detections of very small hotspots in some specific implementations that are expected from the nature of its algorithm, but the level of false detections can be determined to identify the size cut to be applied to the hotspot detection in order to avoid false detections. When the clusters of infections have regular (circular) shapes, both SaTScan and EpiFRIenDs detect them consistently with high statistical significance. However, EpiFRIenDs outperformed SaTScan when the population distributions deviated from circular or elliptical shapes. In these cases, the detection of foci from SaTScan was limited to the areas with densest infections, while EpiFRIenDs correctly identified the whole structures.

Using real data from pregnant women at first ANC visits to identify hotspots, EpiFRIenDs identified hotspots that were adapted to the particular population distributions, while hotspots detected by SaTScan showed again some limitations due to intrinsic shape of its detected hotspots. Adapting to the particularities of the population distributions is a potential of EpiFRIenDs for a more practical use of programmatic activities.

Upcoming feature implementations

The following feature implementations of EpiFRIenDs are currently under development and will be soon released in the next versions for both R and Python:

False detection handling: statistics of false detections will be obtained from simulated runs shuffling the infections of the original data, and the statistical significance of the detected foci will be recalibrated taking into account false positive rates.

Automatisation of linking distance: an iterative process will be run to quantify the output of EpiFRIenDs from different linking distances, proposing the optimal one as the one with more cases belonging to foci weighted by their statistical significance.

Adjusting for local prevalence: different approaches will allow to calibrate the statistical significance of foci taking into account the environmental prevalence instead of the overall prevalence of the data, producing more sensitive detections of small scale fluctuations.

Adaptive linking distance to local density: in order to avoid the different impact of applying the same linking distance in sparse and dense populations simultaneously, an adaptive linking distance will be calculated for each individual taking into account the local population density, using effective larger distances in low-density areas and smaller distances in high-density areas.

User-friendly platform: a user-friendly platform is being developed using RShiny for an easy use without requiring strong technical expertise, and a similar platform will be developed for the Python version.
Visualisation tools: a series of visualisation tools will be developed to produce and visualise the data analysis from the output of EpiFRIenDs.

Final remarks

EpiFRIenDs is a new publicly available software in Python and R to detect epidemiological foci of arbitrary shapes and sizes that are adapted to the population distribution. It is easy to install and use, with documentation of how to install and use it and with Jupyter notebooks with examples of their implementation using the examples from **Supplementary Fig. 7** and reproducing **Supplementary Figs. 8,10,12.**

Supplementary Figures

Supplementary Fig. 1. Comparison of *Plasmodium falciparum* parasite rates between pregnant women with different HIV status and children.

Scatter comparison of *Pf*PR$_{qPCR}$ between pregnant women at all first ANC visits and children 2-10 years old (n=3,933) for (**a**) HIV+ women (n=580), (**b**) HIV- women (n=1,436) and (**c**) primigravid HIV- women (n=532). **d-f** Same as in a-c but for *Pf*PR$_{RDT}$ estimations. **g-i** Same as in D-F but restricted to the low transmission areas (Magude and Manhiça) only (n=3,818, 522, 1,262, 468 for children 2-10 years old, HIV+ women, HIV- women and primigravid HIV- women). Grey lines show the 1-to-1 linear relationship between the two estimations, while the grey dashed line shows the linear regressions of the comparisons. The three points with error bars of each colour represent the mean values for each of the three years of study +/- SD. HIV+: HIV positive; HIV-: HIV negative; PG HIV-: primigravida and HIV negative.

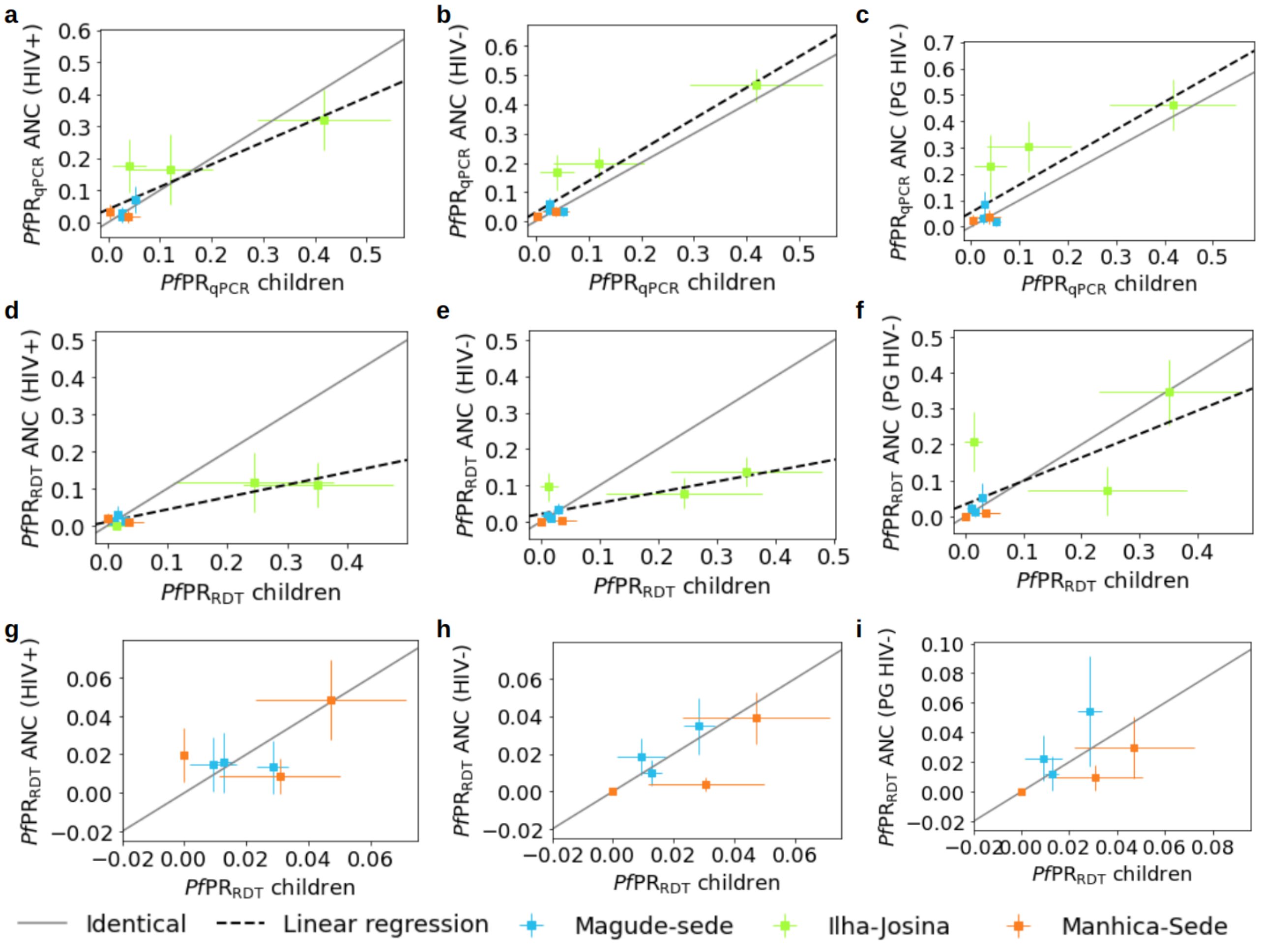

Supplementary Fig. 2. Temporal trends in *Plasmodium falciparum* burden from different data sources.

Comparison of the temporal changes between the number of weekly clinical cases (dashed grey lines) (n=15,467 divided in the three areas) and the positivity rates in children 2-10 years old (n=3,933) from cross-sectional cases (green dots) and in pregnant women at first ANC visits with different selections in parity and HIV status (coloured lines), for the whole study period and for the three studied areas (from top to bottom) (n=1,872, 4,599 and 1,548 for HIV+, HIV- and PG HIV- divided by the three areas). Error bars show the standard deviation of the measurements obtained from Bootstrap resampling. **a, c, e** show results from ANC and cross-sectional data from qPCR results, and **b, d, f** the results estimated for RDT. Time lags were applied to the data corresponding to the values of Table 2 from clinical cases that improve the consistency of the temporal trends. HIV+: HIV positive women; HIV-: HIV negative women; PG HIV-: primigravid HIV negative women. Data are presented as mean values +/- SD.

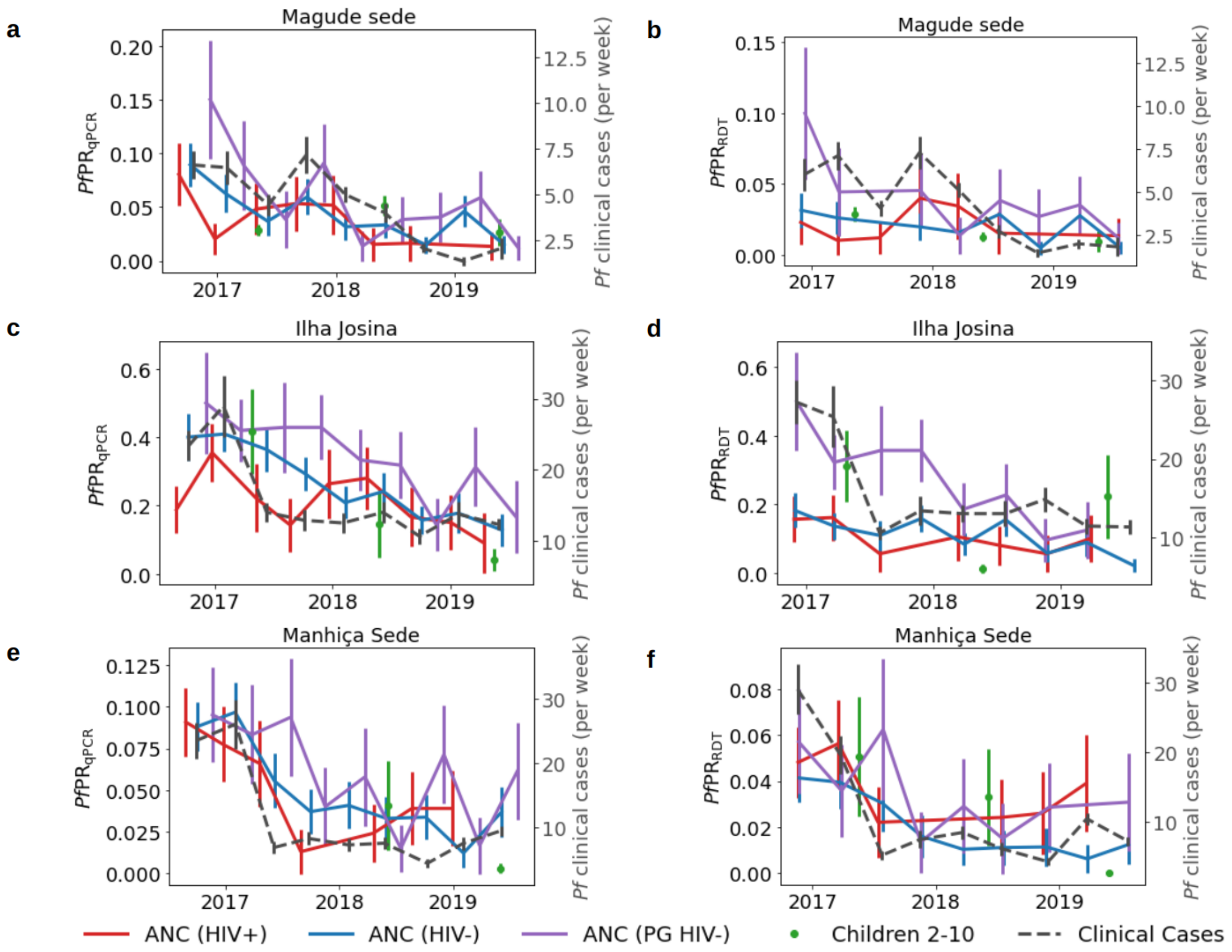

Supplementary Fig. 3. Spatial clustering of *Plasmodium falciparum* infections.

Spatial distribution of qPCR (**a**) and RDT (**b**) confirmed *Plasmodium falciparum* infections at first ANC visit. **c** Spatial distribution of qPCR results in children 2-10 years old from cross-sectional surveys. *Plasmodium falciparum*-positive cases are shown in red and negative in green. **d-f** 2-point correlation functions of RDT-detectable infections in pregnant women at first ANC (different gravidity and HIV status shown in different colours) (n=1,971, 1,613, 1,465 from **d-f**) and in children 2-10 years old (n=1,900, 1,565, 468 from **d-f**) from cross-sectional surveys (black dashed lines) for the three years of study, showing mean values +/- SD. Geographic maps were generated from the public OpenStreetMap data.

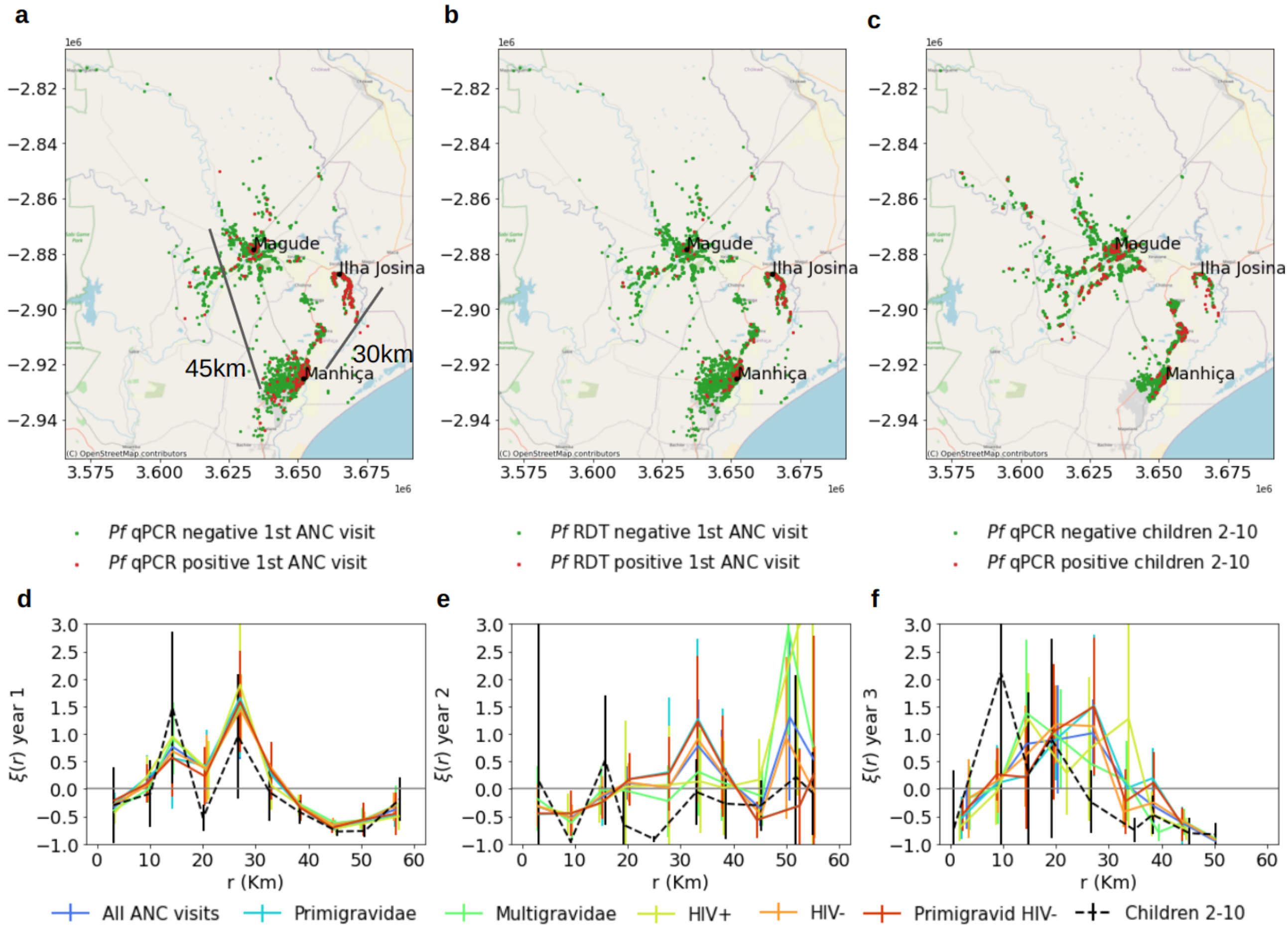

Supplementary Fig. 4. Flowchart of samples analysed with quantitative suspension array assay.

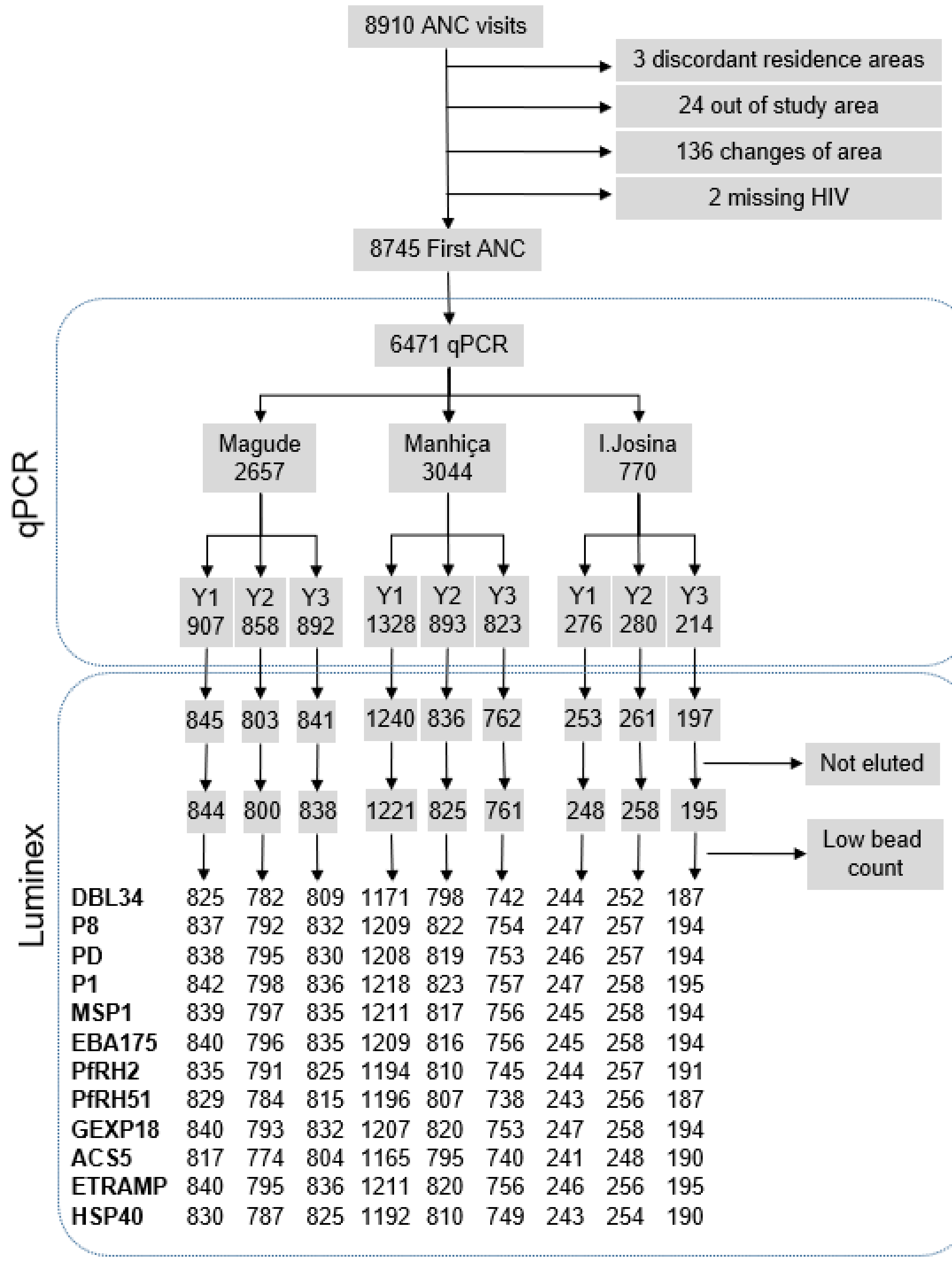

Supplementary Fig. 5. Spatial distribution of *Plasmodium falciparum* hotspots and hotspots of seropositive cases.

a-c Hotspots identified in the three years using qPCR data from first ANC visits. **d-g** Spatial location of hotspots detected using qPCR in children 2-10 years old from cross-sectional surveys (grey) and in pregnant women at first ANC visit (black) and spatial location of sero-clusters detected with different antigens (different colours). **d**, **e** and **g** show a zoom in different sub-regions from **f**. Circles represent detections from year 1, squares show detections from year 2 and triangles from year 3. Geographic maps were generated from the public OpenStreetMap data.

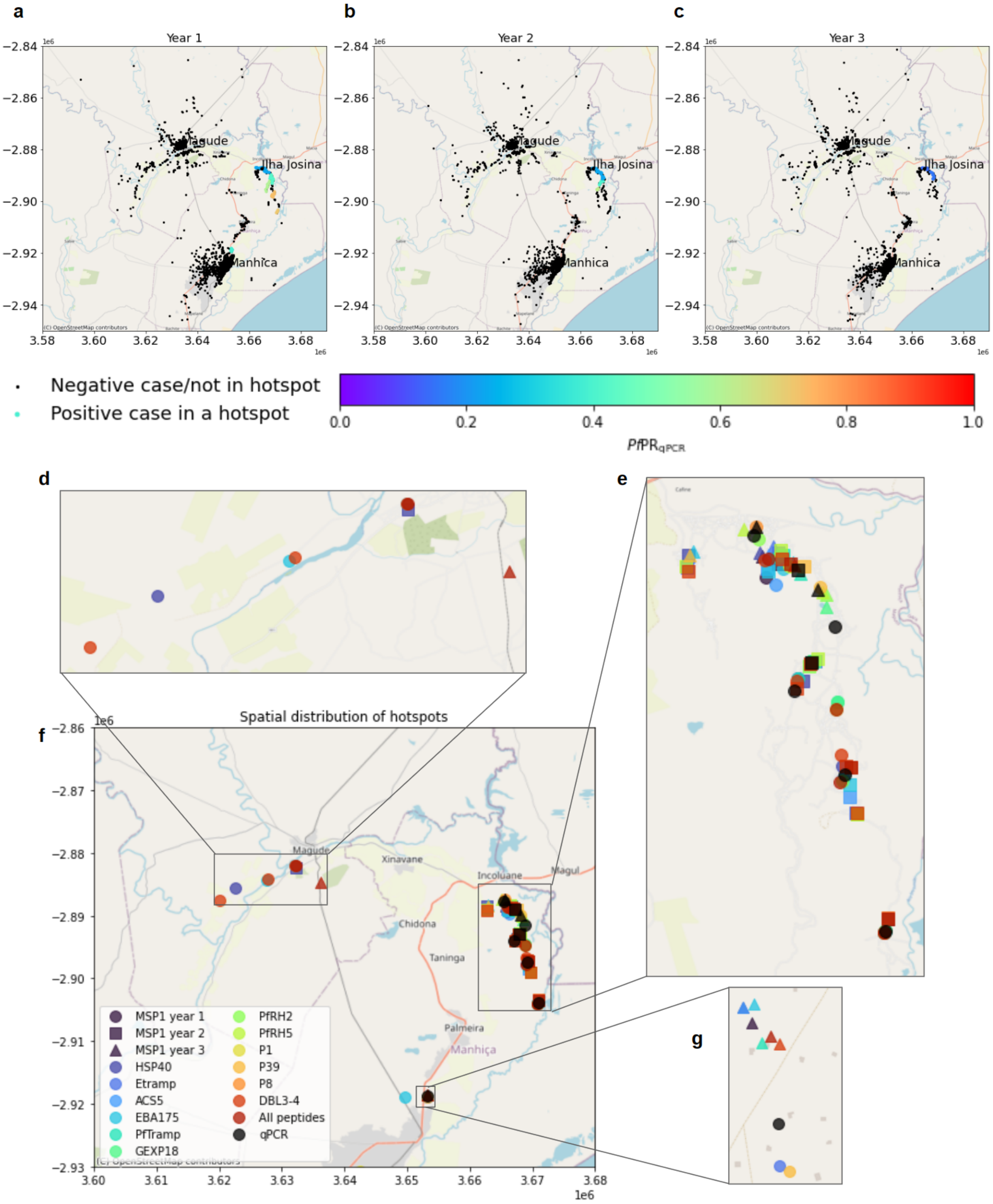

Supplementary Fig. 6. Temporal detection of clusters anti-*Plasmodium falciparum* seropositive cases.

a Temporal distribution of the number of clusters detected. **b** Histogram of lifetimes of identified clusters. **c** Timeline of identified clusters with their size (y-axis) and colour coded by their timeline.

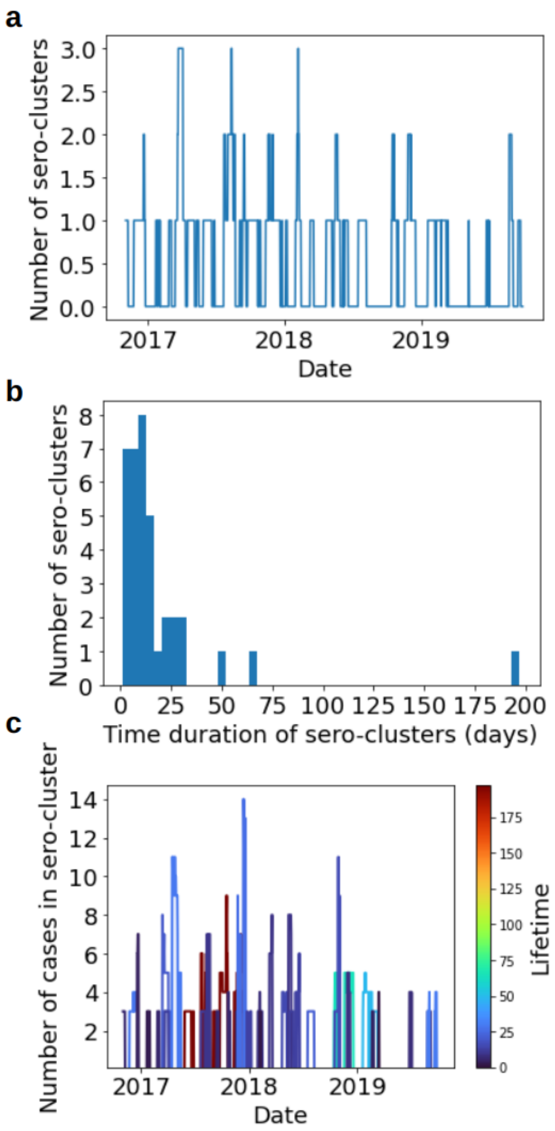

Supplementary Fig. 7. Data generated artificially for testing purposes, showing negative cases in blue and positive cases in red.

a All cases were randomly distributed in space. **b** Negative cases were randomly distributed in space, but positive cases were distributed following four gaussian distributions whose centre was randomly distributed in space. **c** Negative cases were randomly distributed in space, but positive cases were following a sinusoidal distribution.

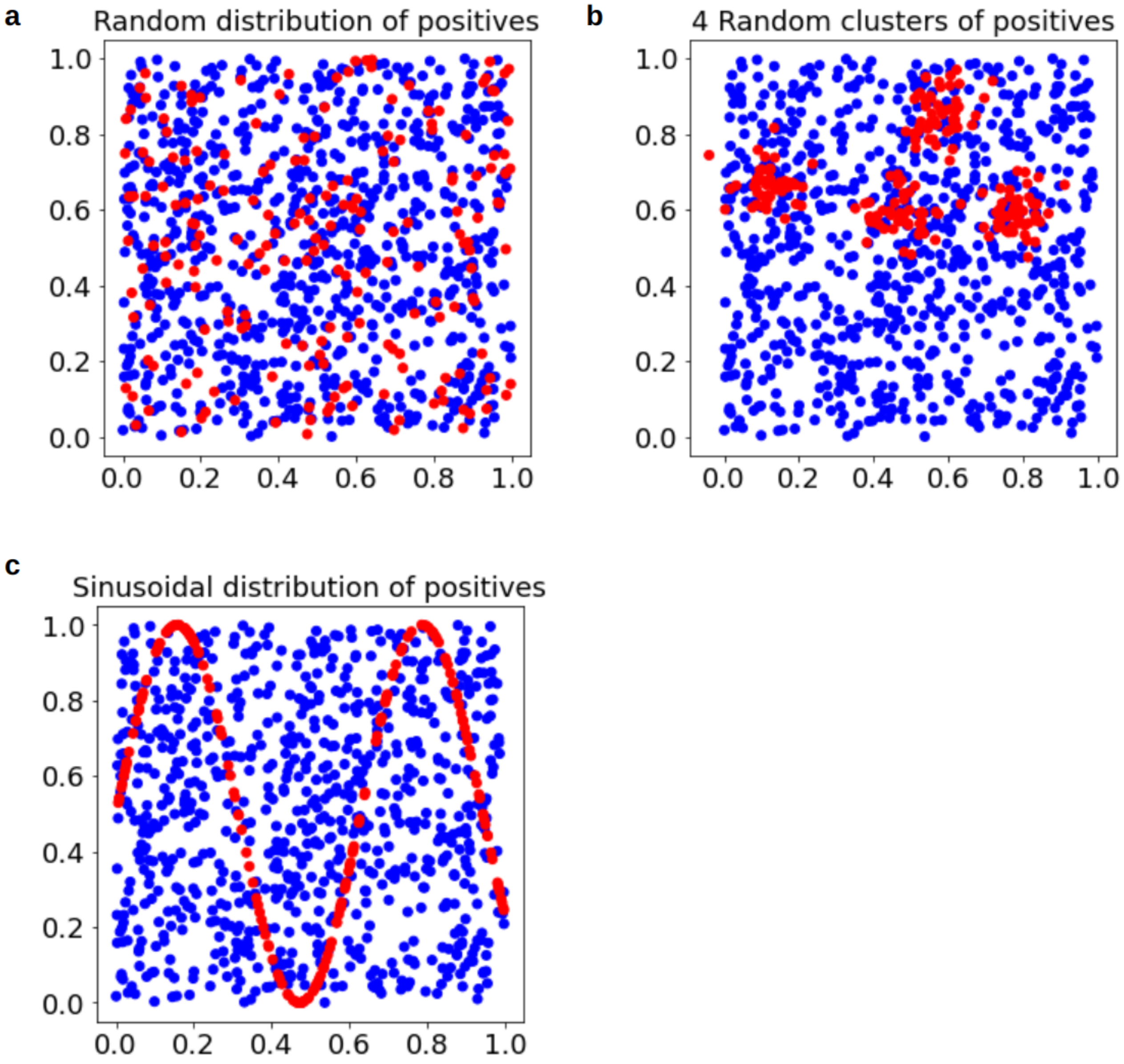

Supplementary Fig. 8. EpiFRIenDs foci detected from random distributions.

P-values of foci detected with EpiFRIenDs for different linking distances in the simulated random distribution. Positive cases belonging to foci are coloured with the p-value of the detection, the rest are shown in grey. All positive cases are circled in red. **a** linking distance = 0.01. **b** linking distance = 0.02. **c** linking distance = 0.05. **d** linking distance = 0.1. Statistical significance was obtained using one-sided t-test statistics, with no adjustments for multiple comparisons.

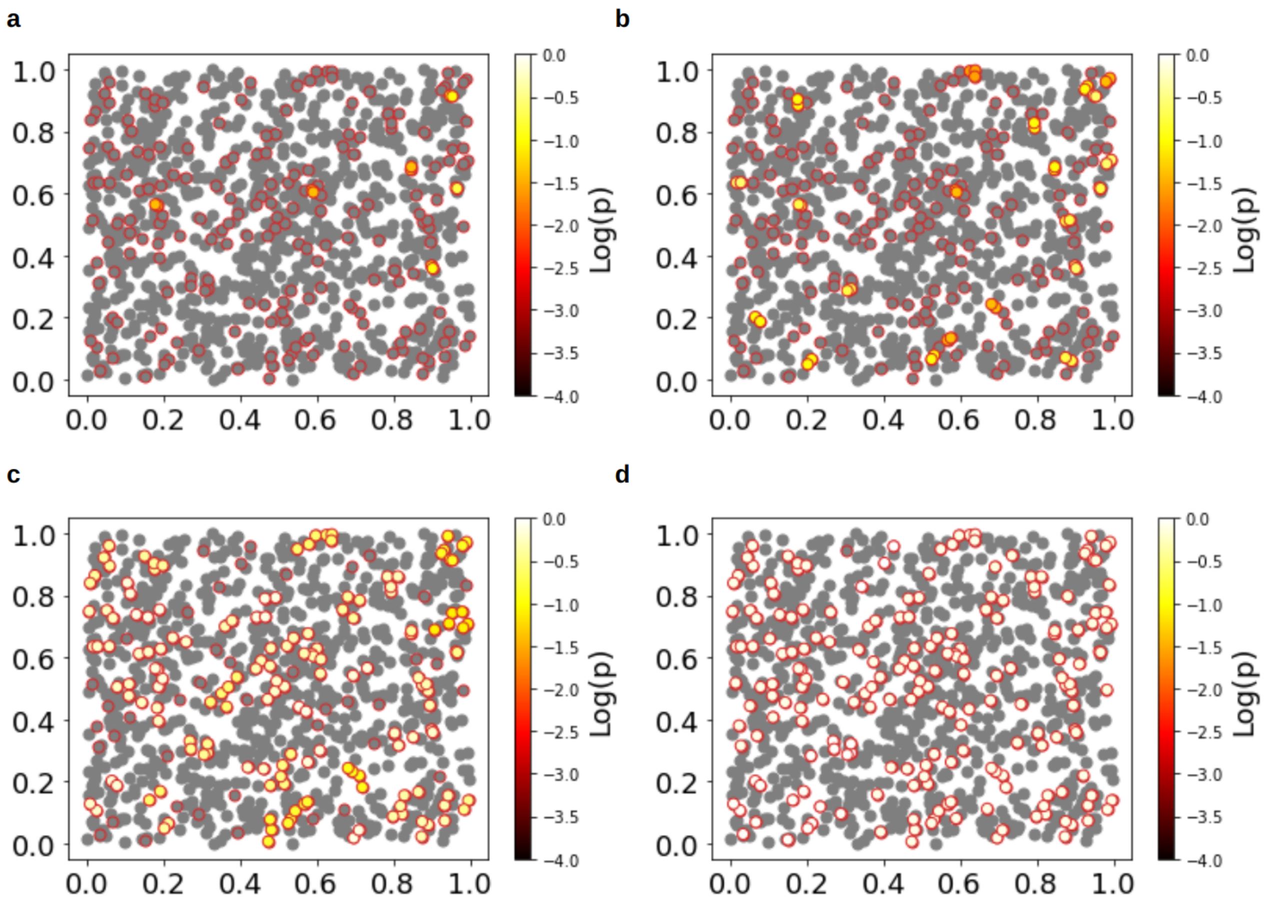

Supplementary Fig. 9. SaTScan foci detected from random distributions.

P-values of foci detected with SaTScan for different linking distances in the simulated random distribution. Positive cases belonging to foci are coloured with the p-value of the detection, the rest are shown in grey. All positive cases are circled in red. **a** maximum window size of 2% of the population. **b** maximum window size of 5% of population. **c** maximum window size of 10% of the population. **d** maximum window size of 30% of the population. Statistical significance was obtained using one-sided t-test statistics, with no adjustments for multiple comparisons.

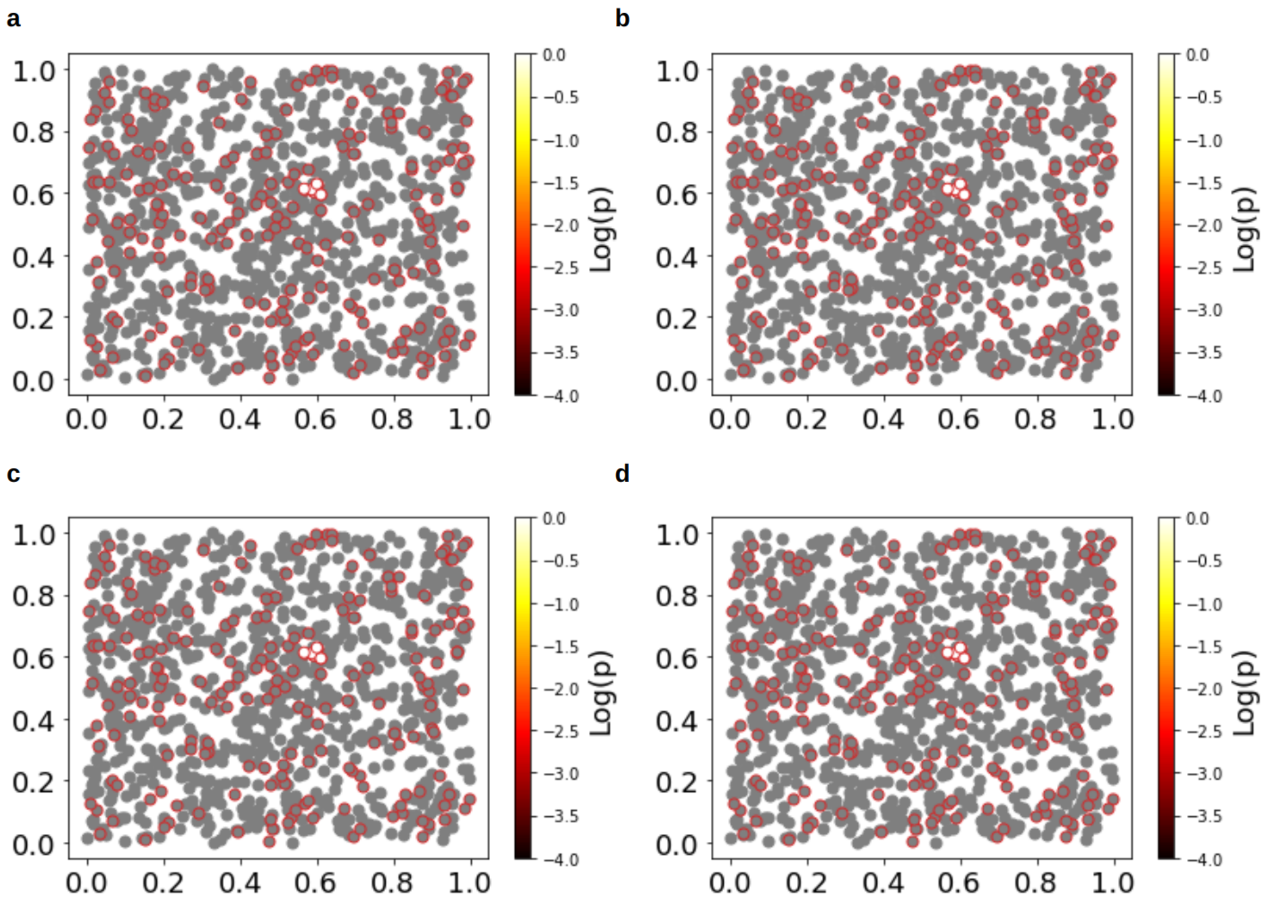

Supplementary Fig. 10. EpiFRIenDs foci detected from clustered distributions.

P-values of foci detected with EpiFRIenDs for different linking distances in the simulated distribution with four clusters. Positive cases belonging to foci are coloured with the p-value of the detection, the rest are shown in grey. All positive cases are circled in red. **a** linking distance = 0.01. **b** linking distance = 0.02. **c** linking distance = 0.05. **d** linking distance = 0.1. Statistical significance was obtained using one-sided t-test statistics, with no adjustments for multiple comparisons.

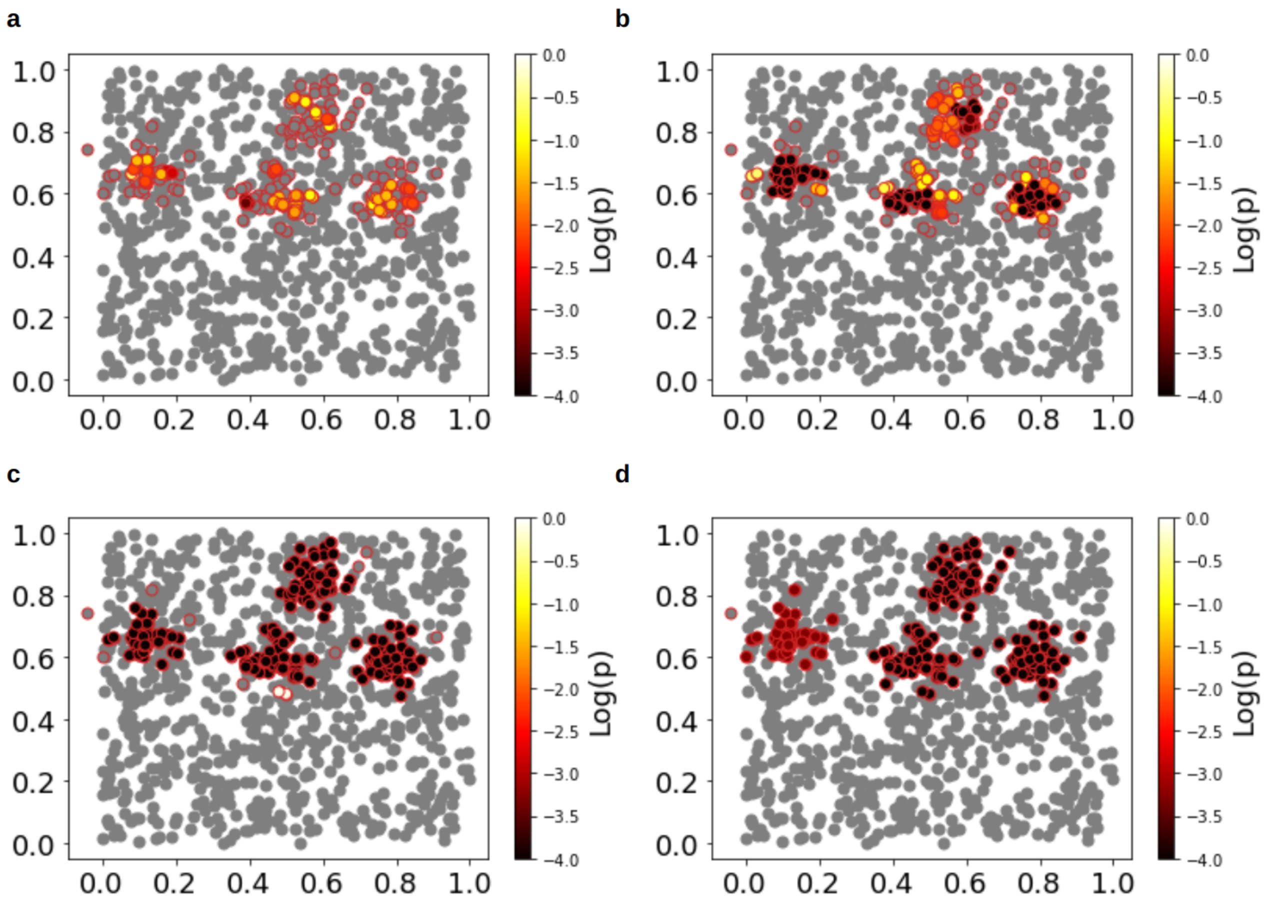

Supplementary Fig. 11. SaTScan foci detected from clustered distributions.

P-values of foci detected with SaTScan for different linking distances in the simulated distribution with four clusters. Positive cases belonging to foci are coloured with the p-value of the detection, the rest are shown in grey. All positive cases are circled in red. **a** maximum window size of 2% of the population. **b** maximum window size of 5% of population. **c** maximum window size of 10% of the population. **d** maximum window size of 30% of the population. Statistical significance was obtained using one-sided t-test statistics, with no adjustments for multiple comparisons.

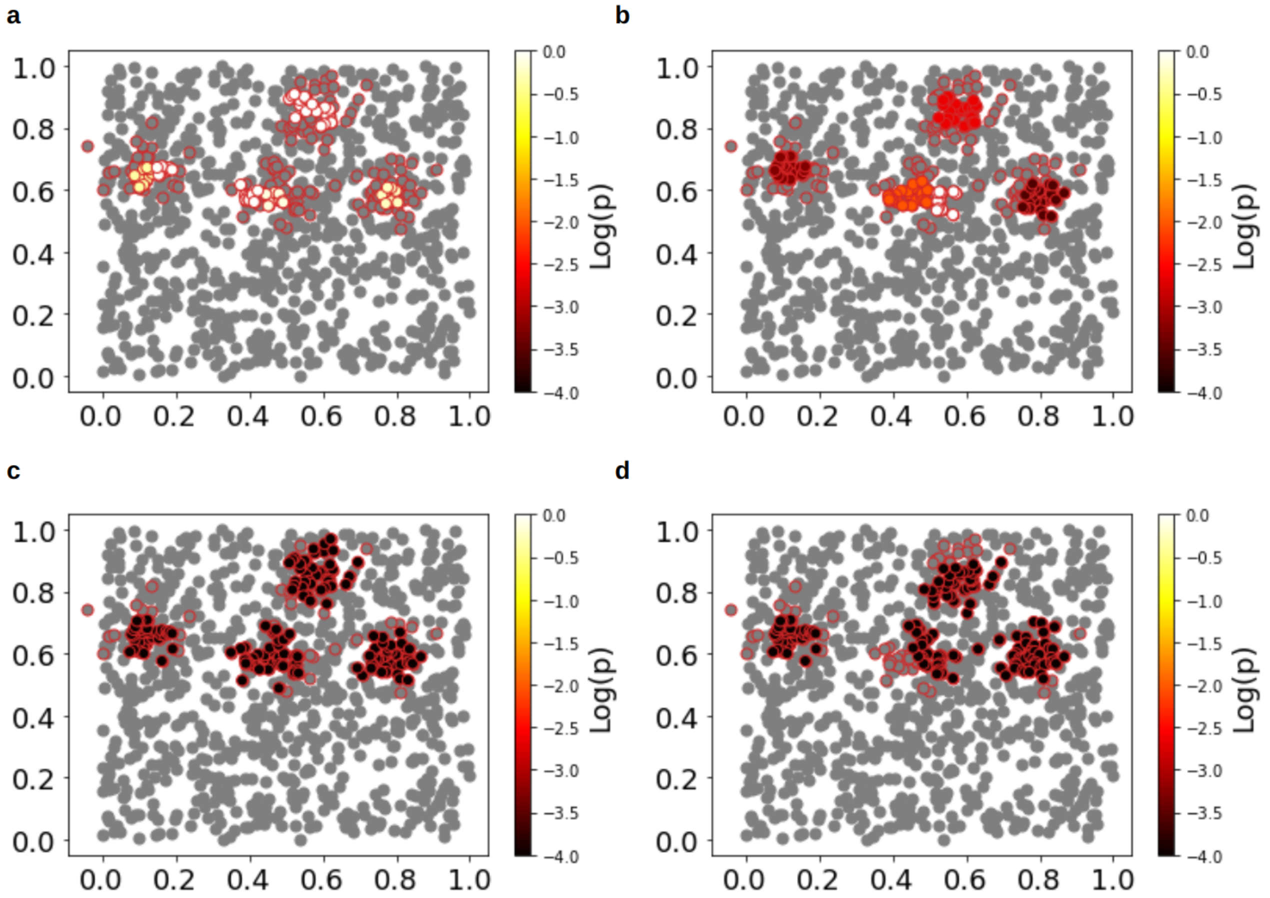

Supplementary Fig. 12. EpiFRIenDs foci detected from sinusoidal distributions.

P-values of foci detected with EpiFRIenDs for different linking distances in the simulated distribution with a sinusoidal distribution of positive cases. Positive cases belonging to foci are coloured with the p-value of the detection, the rest are shown in grey. All positive cases are circled in red. **a** linking distance = 0.01. **b** linking distance = 0.02. **c** linking distance = 0.05. **d** linking distance = 0.1. Statistical significance was obtained using one-sided t-test statistics, with no adjustments for multiple comparisons.

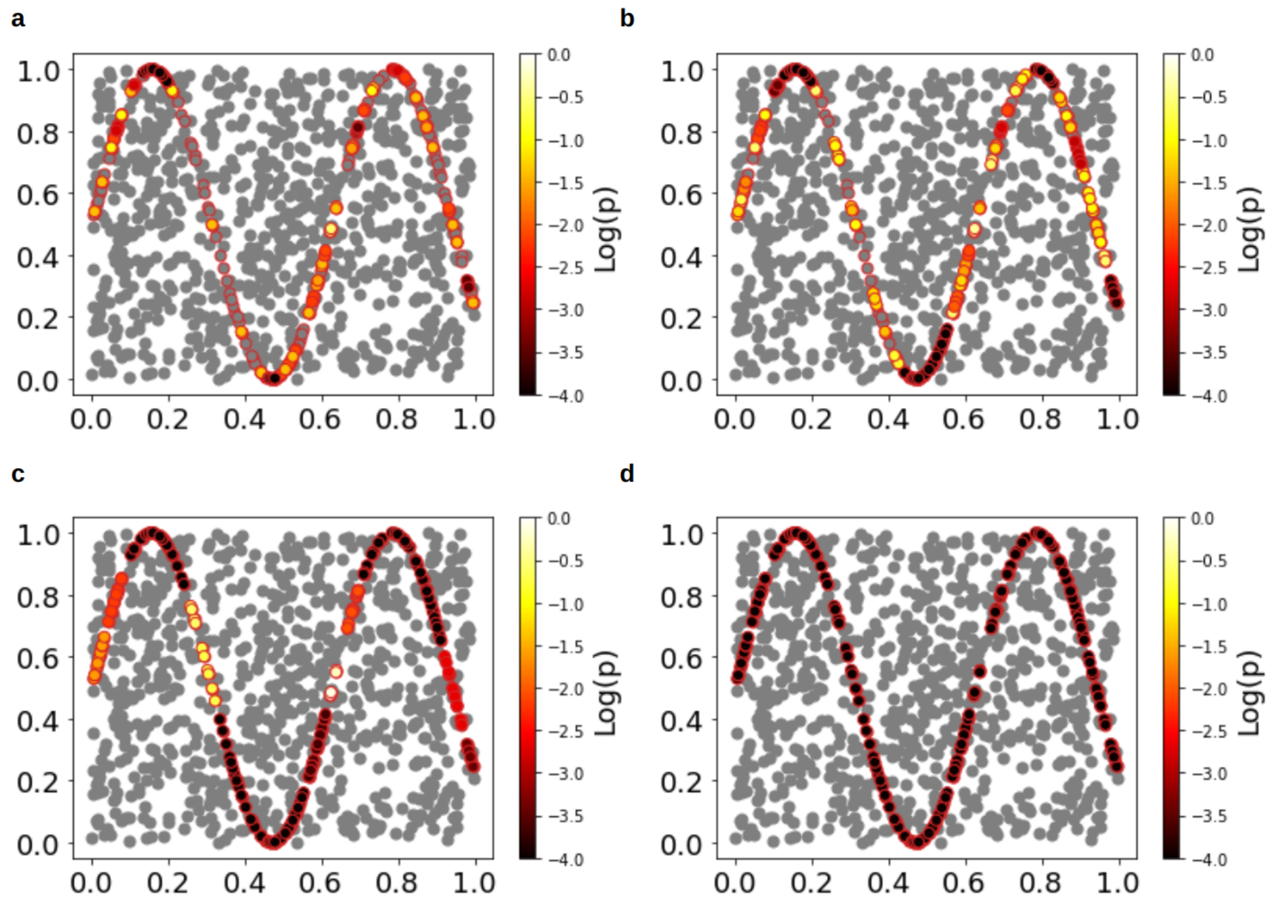

Supplementary Fig. 13. SaTScan foci detected from sinusoidal distributions.

P-values of foci detected with SaTScan for different linking distances in the simulated distribution with a sinusoidal distribution of positive cases. Positive cases belonging to foci are coloured with the p-value of the detection, the rest are shown in grey. All positive cases are circled in red. **a** maximum window size of 2% of the population. **b** maximum window size of 5% of the population. **c** maximum window size of 10% of the population. **d** maximum window size of 30% of the population. Statistical significance was obtained using one-sided t-test statistics, with no adjustments for multiple comparisons.

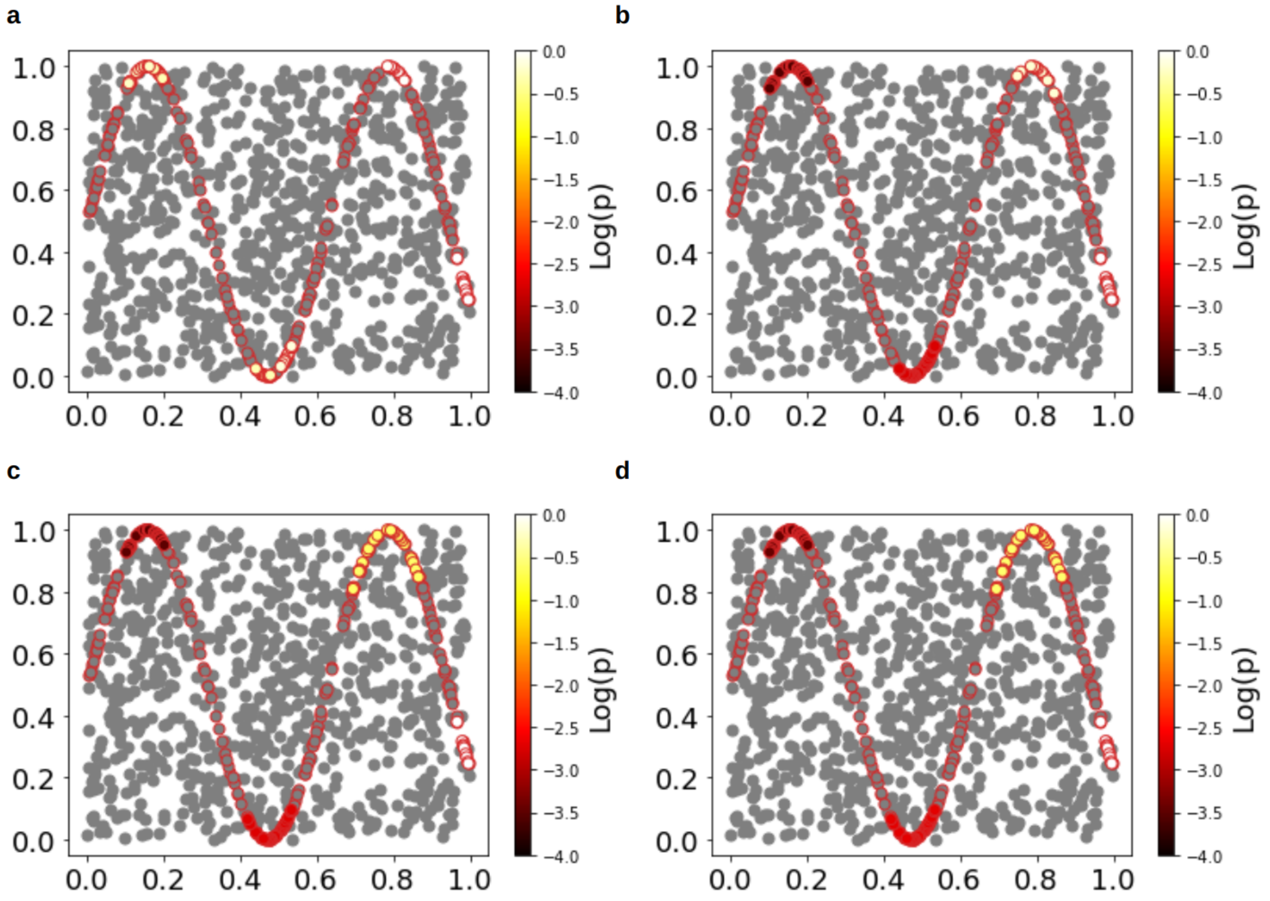

Supplementary Fig. 14. EpiFRIenDs and SaTScan malaria hotspots from real data.

a geographical distribution of households from *Plasmodium falciparum* (*Pf*) qPCR positive (red) and *Pf*-qPCR negative (green) pregnant women attending their first antenatal care (ANC) visit in 2017. **b** the same distribution, but colour coding the positive samples that belong to a hotspot detected by EpiFRIenDs with the positivity rate of the hotspot, and the rest of the samples coloured black. **c** Comparison of the EpiFRIenDs hotspot detector (using a linking distance of 1000m) with SaTScan statistics. Positive samples belonging to an EpiFRIenDs hotspot were coloured according to the *Pf*PR_{PCR} of its hotspot, the rest of them were coloured black. Coloured circles show the hotspots detected by SaTScan with different maximum window sizes. **d** The same as in C, but using a linking distance of 2500m when using EpiFRIenDs. Geographic maps were generated from the public OpenStreetMap data.

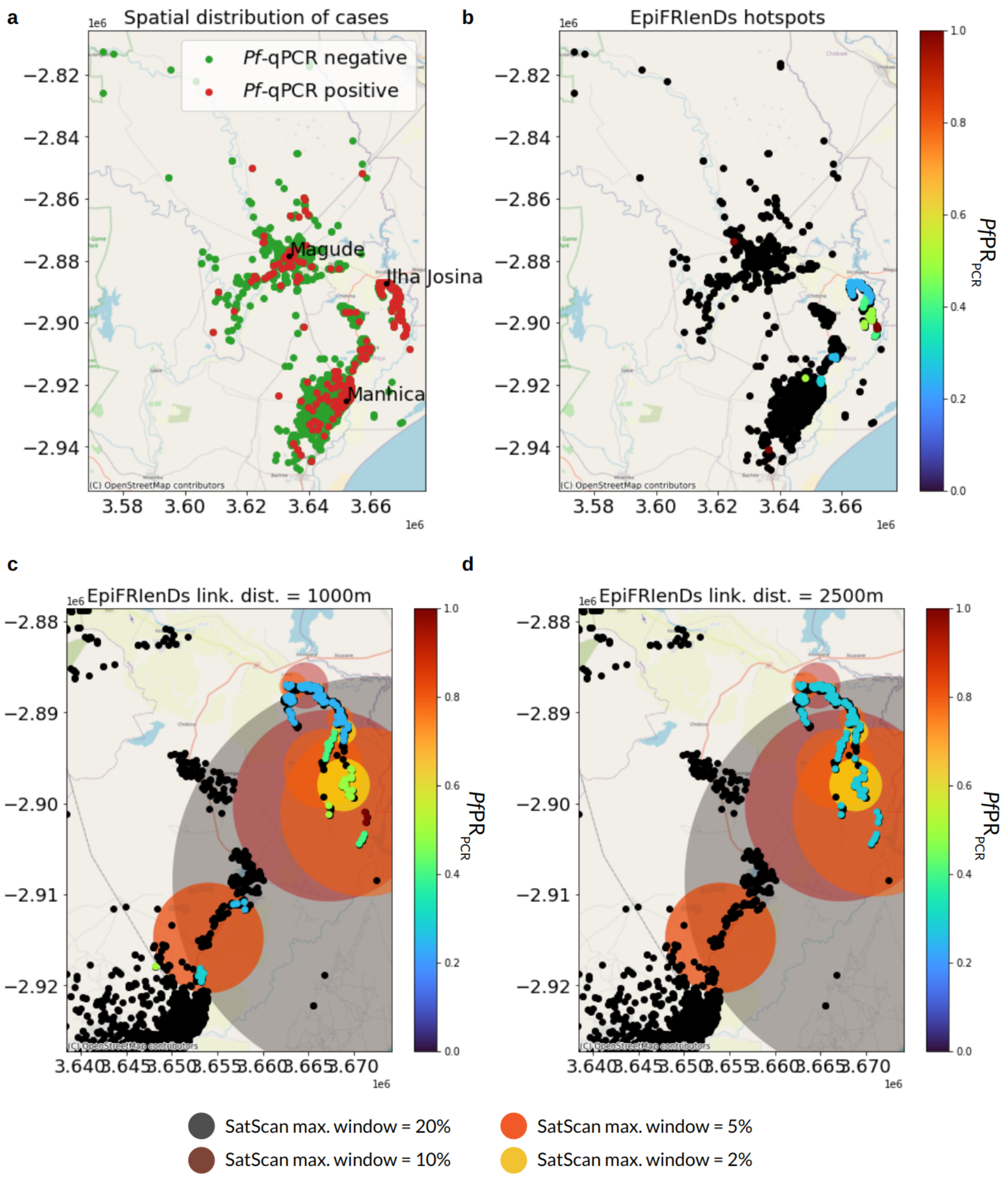

Supplementary Table 1. Correlation between *Plasmodium falciparum* parasite rates between pregnant women at first antenatal care visit and children 2-10 years old.

Parameters of the correlation of $Pf\text{PR}_{qPCR}$ and $Pf\text{PR}_{RDT}$ between pregnant women at first prenatal visits and children 2-10 years old in the three study areas and the three years of study. The slope and origin of the linear regression indicate the linear bias between the two populations and Pearson correlation coefficients indicate the linear correlation between them. The χ^2 statistics was used for the data in Magude and Manhiça sedes (low transmission) only. Consistent relationship between groups is indicated by a slope of 1, origin of 0, Pearson correlation coefficient > 0 and χ^2 < 1.88. 95%CI indicates the 95% confidence interval of each estimate. RDT-based parasite rates among pregnant women were calculated based on parasite densities above 100 parasites/µL.

Test	Population	Slope (95%CI)	Origin (95%CI)	Pearson CC (95%CI)	χ^2 a
qPCR	**All prenatal**	0.97 (0.53, 2.14)	0.03 (-0.01, 0.08)	0.94 (0.70, 0.99)	1.04
	**Primigravidae**	1.14 (0.53, 2.83)	0.05 (-0.02, 0.11)	0.92 (0.53, 0.98)	0.71
	**Multigravidae**	0.89 (0.40, 1.87)	0.03 (-0.01, 0.07)	0.95 (0.67, 0.99)	0.45
	**HIV+**	0.70 (0.20, 1.80)	0.04 (-0.01, 0.10)	0.88 (0.29, 0.98)	0.51
	**HIV-**	1.07 (0.52, 2.33)	0.03 (-0.02, 0.08)	0.95 (0.70, 0.99)	1.05
	**Primigravid HIV-**	1.05 (0.43, 2.52)	0.05 (-0.02, 0.12)	0.86 (0.45, 0.97)	1.02
RDT	**All prenatal**	0.34 (0.11, 0.76)	0.02 (0.01, 0.04)	0.84 (0.31, 0.95)	0.43
	**Primigravidae**	0.74 (0.08, 1.87)	0.04 (-0.00, 0.08)	0.67 (0.09, 0.94)	0.59
	**Multigravidae**	0.17 (-0.04, 0.49)	0.02 (0.00, 0.03)	0.61 (-0.12, 0.94)	0.65
	**HIV+**	0.38 (0.02, 0.89)	0.01 (-0.00, 0.04)	0.96 (0.05, 0.98)	0.44
	**HIV-**	0.32 (0.08, 0.75)	0.03 (0.01, 0.04)	0.76 (0.22, 0.94)	0.53
	**Primigravid HIV-**	0.71 (0.10, 1.80)	0.04 (-0.00, 0.08)	0.66 (0.11, 0.94)	0.46

qPCR: quantitative polymerase chain reaction
RDT: rapid diagnostic test
a: in low transmission settings

Supplementary Table 2. Malaria burden declines from different data sources.

Declines of malaria burden between the first and third years of study (as percentage of reduction) estimated in the three areas from different data sources. P-values were obtained from a two-sided Z-test of proportions.

		Magude			Ilha Josina			Manhiça		
**Test**	**Population**	**PR Year 1 %(95%CI)**	**PR Year 3 %(95%CI)**	**Decline % (p)**	**PR Year 1 %(95%CI)**	**PR Year 3 %(95%CI)**	**Decline % (p)**	**PR Year 1 %(95%CI)**	**PR Year 3 %(95%CI)**	**Decline % (p)**
**qPCR**	**Children 2-10**	2.88 (1.9, 3.95)	2.63 (0.57, 5.33)	8.52 (0.404)	41.7 (15.24, 64.69)	4.03 (0.0, 12.38)	90.33 (0.004)	*	0.3 (0.0, 1.06)	NA
**RDT**		2.88 (1.88, 3.94)	0.94 (0.0, 2.76)	67.49 (0.030)	31.26 (11.79, 52.01)	22.34 (0.0, 47.28)	28.55 (0.282)	5.06 (0.9, 10.66)	0.0 (0.0, 0.0)	100 (NA)
**qPCR**	**All prenatal**	6.39 (4.85, 8.05)	2.25 (1.35, 3.27)	64.78 (0.000)	35.87 (30.07, 41.3)	14.95 (10.28, 20.09)	58.31 (0.000)	8.36 (6.93, 9.86)	3.16 (2.07, 4.38)	62.16 (0.000)
**RDT**		2.09 (1.21, 3.09)	1.01 (0.45, 1.69)	51.62 (0.034)	14.13 (10.14, 18.48)	5.61 (2.8, 8.88)	60.32 (0.001)	3.99 (3.01, 5.05)	1.58 (0.85, 2.43)	60.37 (0.001)
**qPCR**	**Primigravid**	9.89 (5.49, 14.29)	3.9 (1.77, 6.38)	60.56 (0.005)	45.31 (32.81, 57.81)	18.97 (8.62, 29.31)	58.15 (0.001)	10.41 (7.26, 13.88)	5.67 (2.83, 8.91)	45.55 (0.019)
**RDT**		4.95 (2.2, 8.24)	2.13 (0.71, 3.90)	56.97 (0.055)	37.5 (26.56, 50.0)	8.62 (1.72, 17.24)	77.01 (2e-4)	5.99 (3.47, 8.83)	2.83 (0.81, 5.26)	52.72 (0.032)
**MSP1**	**All prenatal**	50.54 (47.2, 53.87)	43.27 (39.9, 46.63)	14.38 (0.002)	77.14 (71.84, 82.45)	70.1 (63.4, 76.29)	9.13 (0.047)	45.83 (42.94, 48.64)	43.58 (40.13, 47.15)	4.92 (0.170)
**EBA175**		37.62 (34.4, 40.83)	31.25 (28.12, 34.38)	16.93 (0.003)	62.04 (55.92, 68.16)	49.48 (42.27, 56.7)	20.24 (0.004)	32.42 (29.78, 35.15)	29.01 (25.83, 32.19)	10.54 (0.054)
**DBL3-4**		29.7 (26.67, 32.73)	21.09 (18.36, 23.95)	28.98 (1e-4)	58.61 (52.46, 64.75)	44.92 (37.97, 51.87)	23.35 (0.003)	21.01 (18.62, 23.31)	16.04 (13.48, 18.73)	23.66 (0.003)
**P1**		9.38 (7.48, 11.4)	6.84 (5.16, 8.64)	27.07 (0.028)	16.6 (12.15, 21.46)	11.79 (7.69, 16.41)	28.94 (0.073)	13.14 (11.33, 15.11)	8.99 (7.01, 10.98)	31.53 (0.002)
**P39**		7.81 (6.01, 9.74)	8.34 (6.5, 10.31)	-6.8 (0.647)	18.78 (13.88, 23.67)	12.9 (8.06, 18.28)	31.28 (0.047)	8.48 (6.91, 10.04)	8.22 (6.33, 10.24)	3.02 (0.419)
**P8**		6.93 (5.26, 8.72)	5.55 (4.1, 7.12)	19.92 (0.118)	15.79 (11.34, 20.24)	9.79 (5.67, 13.92)	37.97 (0.032)	5.62 (4.38, 6.95)	6.51 (4.78, 8.37)	-15.7 (0.781)
**PD**		11.22 (9.19, 13.37)	9.07 (7.13, 11.12)	19.15 (0.078)	18.29 (13.41, 23.17)	11.86 (7.22, 16.49)	35.19 (0.027)	12.33 (10.51, 14.24)	9.44 (7.45, 11.57)	23.45 (0.021)
**All peptides**		20.71 (18.0, 23.41)	19.02 (16.39, 21.65)	8.15 (0.187)	34.4 (28.4, 40.4)	25.76 (19.7, 31.82)	25.12 (0.024)	22.83 (20.55, 25.1)	19.95 (17.19, 22.83)	12.61 (0.067)
**DBL3-4**	**Primigravid**	13.25 (8.43, 18.67)	5.95 (3.17, 9.13)	55.09 (0.007)	36.0 (24.0, 50.0)	24.07 (12.96, 35.19)	33.13 (0.086)	13.82 (9.82, 18.18)	8.26 (5.05, 11.93)	40.25 (0.024)
		**Weekly av. cases Year 1 (95%CI)**	**Weekly av. cases Year 3 (95%CI)**	**Decline % (p)**	**Weekly av. cases Year 1 (95%CI)**	**Weekly av. cases Year 3 (95%CI)**	**Decline % (p)**	**Weekly av. cases Year 1 (95%CI)**	**Weekly av. cases Year 3 (95%CI)**	**Decline % (p)**
**RDT**	**Clinical cases**	6.04 (5.04, 7.06)	1.65 (1.27, 2.13)	72.61 (0.000)	20.02 (16.4,2 24.02)	12.38 (10.98, 13.86)	38.16 (0.000)	16.42 (12.60, 20.66)	7.81 (6.81, 8.85)	52.44 (0.000)

Supplementary Table 3. Consistency of 2-point correlation function of *Plasmodium falciparum* infections between children and pregnant women at antenatal care.

χ2 statistics comparing the 2PCF outcomes of *Plasmodium falciparum* infections detected by qPCR in children 2-10 years old with those from different populations of pregnant women at first ANC visit.

Test	Population	Year 1	Year 2	Year 3
qPCR	**All ANC visits**	0.53	1.46	0.70
	**Primigravidae**	0.35	0.68	0.68
	**Multigravidae**	0.33	1.53	0.61
	**HIV+**	0.41	0.68	0.52
	**HIV-**	0.31	1.33	0.70
	**Primigravid HIV-**	0.56	0.62	0.59
RDT	**All ANC visits**	0.41	0.63	0.57
	**Primigravidae**	0.44	0.72	0.65
	**Multigravidae**	0.46	0.73	0.66
	**HIV+**	0.42	0.44	0.60
	**HIV-**	0.52	0.72	0.44
	**Primigravid HIV-**	0.38	0.57	0.59

Supplementary Table 4. Statistical significance of clustering signal.

P-value of statistical significance of clustering signal (2PCF measurements deviating from 0) for different data sources and populations. P-values were obtained from a two-sided Z-test of proportions.

Test	Population	Year 1	Year 2	Year 3
**qPCR**	**Children 2-10**	5.8e-7	0.00	0.33
**RDT**	**Children 2-10**	0.00	0.00	3.1e-5
**qPCR**	**All ANC visits**	0.00	1.6e-4	0.25
	**Primigravidae**	5.2e-7	0.01	0.43
	**Multigravidae**	0.00	2.1e-5	0.10
	**HIV+**	0.00	0.08	0.82
	**HIV-**	0.00	4.8e-5	0.19
	**Primigravid HIV-**	0.00	0.01	0.75
**RDT**	**All ANC visits**	0.00	0.56	0.00
	**Primigravidae**	0.00	0.57	0.00
	**Multigravidae**	3.4e-6	0.01	2.7e-7
	**HIV+**	0.00	1.00	3.5e-5
	**HIV-**	0.00	0.26	0.00
	**Primigravid HIV-**	0.00	0.78	0.00

Supplementary Table 5. Fraction of hostpots spatially and spatiotemporally matched between ANC and clinical data.

Total number of hotspots detected using EpiFRIenDs using the two data sources (ANC and OPMSS), and the fraction of hotspots that matched between the two data sources takin into account only their proximity in space (spatially matched) and taking into account their proximity in both space and time (spatio-temporally matched).

Data source	Total detected	Spatially matched	Spatio-temporally matched
ANC	11	9 (81.81%)	7 (63.63%)
OPMSS	14	9 (64.29%)	7 (50.00%)

Supplementary Table 6. Number of detected *Plasmodium falciparum* hotspots and clusters of seropositive cases.

Number of hotspots in the three years of study from pregnant women at first ANC visit (for qPCR and RDT and different parity and HIV status selections) and number of sero-clusters detected from the different antibodies. Since not all the qPCR samples of pregnant women have serological data, we also show the number of hotspots found with qPCR data restricting the analysis to the samples with serology data, so that the same spatial distribution and densities are used to identify hotspots and sero-clusters for a fair comparison.

Test	Population	Year 1	Year 2	Year 3
qPCR	All ANC visits	6	3	2
	Primigravidae	2	1	0
	Multigravidae	5	1	1
	HIV+	0	0	0
	HIV-	5	3	2
	Primigravida HIV-	2	1	0
RDT	All ANC visits	3	1	0
	Primigravidae	2	1	0
	Multigravidae	0	0	0
	HIV+	0	0	0
	HIV-	2	1	0
	Primigravida HIV-	0	1	0
qPCR	First ANC visits serology sample	6	2	2
MSP1	First ANC visits	2	2	2
HSP40		6	5	2
Etramp		4	4	2
ACS5		3	2	2
EBA175		6	2	3
PfTramp		4	2	2
GEXP18		2	1	1
PfRH2		3	2	1
PfRH5		3	4	2
P1		1	0	0
P39		2	2	1
P8		1	0	0
PD		0	2	0
DBL3-4		7	5	2
Any peptide		5	4	2

Supplementary Table 7. Seroprevalence of anti-*Plasmodium falciparum* antibodies among pregnant women at first antenatal care visit.

Seroprevalence, i.e. percentage seropositive, for each antigen in year 1-3 in Magude, Manhica and Ilha Josina with 95% confidence intervals.

	**Magude**			**Manhica**			**Ilha Josina**		
	**Y1**	**Y2**	**Y3**	**Y1**	**Y2**	**Y3**	**Y1**	**Y2**	**Y3**
**MSP1**	50.5% (47.1-54)	46.5% (43-50.1)	43.2% (39.9-46.7)	45.8% (43-48.7)	42.7% (39.3-46.2)	43.5% (40-47.1)	77.1% (71.3-82.1)	72.5% (66.5-77.7)	70.1% (63.1-76.3)
**HSP40**	21.4% (18.7-24.4)	17.2% (14.6-20)	13.9% (11.7-16.5)	16.7% (14.6-19)	13.7% (11.4-16.3)	14% (11.7-16.8)	39.9% (33.8-46.4)	47.6% (41.4-54)	41.1% (34.1-48.4)
**Etramp**	17.0% (14.6-19.8)	15.6% (13.2-18.4)	12.6% (10.4-15)	15.7% (13.7-17.9)	12.9% (10.7-15.5)	13.5% (11.2-16.2)	37.4% (31.4-43.8)	38.7% (32.7-45)	28.7% (22.6-35.7)
**ACS5**	34.4% (31.2-37.8)	28.8% (25.7-32.2)	26.9% (23.9-30.1)	26.4% (23.9-29)	22.6% (19.8-25.7)	23% (20-26.2)	60.6% (54.1-66.7)	62.5% (56.1-68.5)	55.3% (47.9-62.4)
**EBA175**	37.6% (34.3-41)	33.4% (30.2-36.8)	31.3% (28.1-34.5)	32.4% (29.8-35.2)	29.0% (26-32.3)	29.0% (25.8-32.4)	62.0% (55.6-68.1)	55.8% (49.5-61.9)	49.5% (42.3-56.7)
**PfTramp**	21.0% (18.4-24)	21.7% (18.9-24.8)	17.0% (14.5-19.7)	14.8% (12.8-16.9)	13.8% (11.5-16.4)	15.6% (13.1-18.4)	35.8% (29.9-42.1)	34.1% (28.4-40.3)	30.4% (24.1-37.5)
**GEXP18**	9.4% (7.6-11.6)	5.9% (4.4-7.9)	6.0% (4.5-7.9)	8.0% (6.6-9.8)	5.4% (4-7.2)	7.2% (5.5-9.3)	19.8% (15.2-25.5)	15.5% (11.4-20.6)	12.4% (8.2-18)
**PfRH2**	13.8% (11.5-16.3)	12.4% (10.2-14.9)	9.9% (8-12.2)	13.9% (12-16)	10.7% (8.7-13.1)	12.2% (10-14.8)	29.5% (23.9-35.7)	25.3% (20.2-31.1)	20.9% (15.5-27.5)
**PfRH5**	16.0% (13.6-18.8)	14.9% (12.5-17.7)	11.8% (9.7-14.2)	14.7% (12.8-16.9)	11.2% (9.1-13.6)	11.2% (9.1-13.8)	35.8% (29.8-42.2)	34.4% (28.6-40.6)	29.4% (23.1-36.6)
**P1**	9.4% (7.5-11.6)	8.6% (6.8-10.9)	6.8% (5.2-8.8)	13.1% (11.3-15.2)	8.1% (6.4-10.3)	9.0% (7.1-11.3)	16.6% (12.3-22)	16.3% (12.1-21.5)	11.8% (7.8-17.4)
**P39**	7.8% (6.1-9.9)	8.2% (6.4-10.4)	8.3% (6.6-10.5)	8.5% (7-10.2)	6.6% (5.1-8.6)	8.2% (6.4-10.5)	18.8% (14.2-24.4)	22.4% (17.5-28.1)	12.9% (8.6-18.8)
**P8**	6.9% (5.3-8.9)	7.2% (5.5-9.3)	5.5% (4.1-7.4)	5.6% (4.4-7.1)	5.6% (4.2-7.5)	6.5% (4.9-8.6)	15.8% (11.6-21.1)	16% (11.8-21.1)	9.8% (6.2-15.1)
**PD**	11.2% (9.2-13.6)	11.8% (9.7-14.3)	9.0% (7.2-11.2)	12.3% (10.6-14.4)	9.6% (7.8-11.9)	9.4% (7.5-11.8)	18.3% (13.8-23.8)	19.5% (14.9-24.9)	11.9% (7.8-17.5)
**DBL3-4**	29.7% (26.6-33)	24.2% (21.2-27.4)	21.0% (18.3-24)	21.0% (18.7-23.5)	16.9% (14.4-19.7)	16.0% (13.5-18.9)	58.6% (52.1-64.8)	54.4% (48-60.6)	44.9% (37.7-52.3)

Supplementary Table 8. Correlation between *Plasmodium falciparum* parasite rates in children 2-10 years old and anti-*Plasmodium falciparum* seroprevalence in pregnant women at first antenatal care visit.

Parameters of the correlation of *Pf*PR$_{qPCR}$ in children 2-10 years old and seroprevalence in pregnant women in the three study areas and the three years of study. The slope and origin of the linear regression indicate the linear bias between the two populations and Pearson CC indicates the linear correlation between them. 95%CI indicates the 95% confidence interval of each estimate.

	Slope (95%CI)	Origin (95%CI)	Pearson CC (95%CI)
**MSP1**	1.05 (0.55, 2.06)	0.45 (0.40, 0.51)	0.79 (0.48, 0.91)
**HSP40**	0.85 (0.36, 2.03)	0.17 (0.11, 0.22)	0.73 (0.37, 0.91)
**Etramp**	0.83 (0.38, 1.77)	0.16 (0.11, 0.21)	0.81 (0.43, 0.93)
**ACS5**	1.24 (0.65, 2.54)	0.26 (0.20, 0.32)	0.78 (0.48, 0.90)
**EBA175**	0.91 (0.48, 2.23)	0.31 (0.26, 0.36)	0.84 (0.51, 0.94)
**PfTramp**	0.54 (0.19, 1.22)	0.19 (0.15, 0.23)	0.70 (0.28, 0.89)
**GEXP18**	0.56 (0.25, 1.15)	0.05 (0.02, 0.08)	0.94 (0.51, 0.97)
**PfRH2**	0.71 (0.31, 1.47)	0.10 (0.07, 0.14)	0.93 (0.53, 0.96)
**PfRH5**	1.02 (0.51, 2.15)	0.11 (0.06, 0.16)	0.91 (0.56, 0.97)
**P1**	0.28 (0.05, 0.69)	0.08 (0.06, 0.11)	0.83 (0.14, 0.92)
**P39**	0.37 (0.09, 0.90)	0.08 (0.05, 0.11)	0.87 (0.21, 0.95)
**P8**	0.45 (0.18, 0.95)	0.05 (0.03, 0.08)	0.89 (0.45, 0.94)
**PD**	0.37 (0.09, 0.79)	0.08 (0.06, 0.11)	0.90 (0.29, 0.95)
**DBL3-4**	1.10 (0.55, 2.52)	0.20 (0.15, 0.26)	0.88 (0.56, 0.97)
**Any peptide**	0.58 (0.22, 1.26)	0.20 (0.16, 0.24)	0.83 (0.31, 0.92)

Supplementary Table 9. Declines in seroprevalence of anti-*Plasmodium falciparum* antibodies among pregnant women at first antenatal care visit.

Declines of anti-*Plasmodium falciparum* seroprevalence between the first and third years of study (as percentage of reduction) estimated in the three areas from different antibodies. P-values were obtained from a two-sided Z-test of proportions. All: all first ANC visits; PG: primigravid women

		**Magude**			**Ilha Josina**			**Manhiça**		
**Data**	**Antigen**	**Year 1 %(95%CI)**	**Year 3 %(95%CI)**	**Decline % (p)**	**Year 1 %(95%CI)**	**Year 3 %(95%CI)**	**Decline % (p)**	**Year 1 %(95%CI)**	**Year 3 %(95%CI)**	**Decline % (p)**
All	**MSP1**	50.54 (47.2, 53.87)	43.27 (39.9, 46.63)	14.38 (0.002)	77.14 (71.84, 82.45)	70.1 (63.4, 76.29)	9.13 (0.047)	45.83 (42.94, 48.64)	43.58 (40.13, 47.15)	4.92 (0.170)
	**HSP40**	21.45 (18.67, 24.22)	13.87 (11.56, 16.3)	35.33 (0.0001)	39.92 (33.74, 46.09)	41.05 (34.21, 48.42)	-2.84 (0.596)	16.69 (14.6, 18.79)	14.04 (11.63, 16.58)	15.92 (0.053)
	**Etramp**	17.02 (14.52, 19.64)	12.61 (10.32, 14.89)	25.96 (0.005)	37.4 (31.3, 43.09)	28.72 (22.56, 35.38)	23.21 (0.028)	15.69 (13.71, 17.75)	13.51 (11.13, 16.03)	13.89 (0.089)
	**ACS5**	34.39 (31.09, 37.7)	26.81 (23.82, 29.93)	22.06 (0.001)	60.58 (54.36, 66.8)	55.26 (47.89, 62.11)	8.78 (0.134)	26.35 (23.86, 28.93)	22.97 (20.0, 25.95)	12.82 (0.045)
	**EBA175**	37.62 (34.4, 40.83)	31.25 (28.12, 34.38)	16.93 (0.003)	62.04 (55.92, 68.16)	49.48 (42.27, 56.7)	20.24 (0.004)	32.42 (29.78, 35.15)	29.01 (25.83, 32.19)	10.54 (0.054)
	**PfTramp**	21.05 (18.31, 23.78)	17.05 (14.53, 19.69)	19.0 (0.018)	35.77 (30.08, 41.87)	30.41 (24.23, 36.6)	14.98 (0.115)	14.76 (12.86, 16.82)	15.61 (13.1, 18.25)	-5.77 (0.670)
	**GEXP18**	9.4 (7.5, 11.43)	6.03 (4.46, 7.72)	35.87 (0.005)	19.84 (14.98, 25.1)	12.37 (7.73, 17.01)	37.64 (0.017)	8.04 (6.55, 9.61)	7.18 (5.45, 9.04)	10.65 (0.243)
	**PfRH2**	13.77 (11.5, 16.17)	9.98 (8.03, 12.04)	27.57 (0.008)	29.51 (23.77, 35.25)	20.94 (15.18, 26.7)	29.03 (0.018)	13.9 (11.98, 15.91)	12.23 (9.95, 14.65)	12.02 (0.137)
	**PfRH5**	16.04 (13.51, 18.58)	11.82 (9.61, 14.16)	26.31 (0.007)	35.8 (30.04, 41.98)	29.41 (22.99, 35.83)	17.85 (0.078)	14.72 (12.71, 16.72)	11.26 (9.09, 13.57)	23.47 (0.016)
	**P1**	9.38 (7.48, 11.4)	6.84 (5.16, 8.64)	27.07 (0.028)	16.6 (12.15, 21.46)	11.79 (7.69, 16.41)	28.94 (0.073)	13.14 (11.33, 15.11)	8.99 (7.01, 10.98)	31.53 (0.002)
	**P39**	7.81 (6.01, 9.74)	8.34 (6.5, 10.31)	-6.8 (0.647)	18.78 (13.88, 23.67)	12.9 (8.06, 18.28)	31.28 (0.047)	8.48 (6.91, 10.04)	8.22 (6.33, 10.24)	3.02 (0.419)
	**P8**	6.93 (5.26, 8.72)	5.55 (4.1, 7.12)	19.92 (0.118)	15.79 (11.34, 20.24)	9.79 (5.67, 13.92)	37.97 (0.032)	5.62 (4.38, 6.95)	6.51 (4.78, 8.37)	-15.7 (0.781)
	**PD**	11.22 (9.19, 13.37)	9.07 (7.13, 11.12)	19.15 (0.078)	18.29 (13.41, 23.17)	11.86 (7.22, 16.49)	35.19 (0.027)	12.33 (10.51, 14.24)	9.44 (7.45, 11.57)	23.45 (0.021)
	**DBL3-4**	29.7 (26.67, 32.73)	21.09 (18.36, 23.95)	28.98 (0.0001)	58.61 (52.46, 64.75)	44.92 (37.97, 51.87)	23.35 (0.003)	21.01 (18.62, 23.31)	16.04 (13.48, 18.73)	23.66 (0.003)
	**All peptides**	20.71 (18.0, 23.41)	19.02 (16.39, 21.65)	8.15 (0.187)	34.4 (28.4, 40.4)	25.76 (19.7, 31.82)	25.12 (0.024)	22.83 (20.55, 25.1)	19.95 (17.19, 22.83)	12.61 (0.067)
PG	**MSP1**	48.81 (41.07, 56.55)	41.22 (35.11, 46.95)	15.55 (0.062)	72.55 (60.78, 84.31)	66.07 (53.57, 78.57)	8.93 (0.238)	43.55 (37.97, 49.48)	43.05 (36.32, 49.33)	1.16 (0.448)
	**HSP40**	21.21 (15.15, 27.88)	11.97 (8.11, 16.22)	43.57 (0.005)	42.0 (28.0, 56.0)	41.82 (29.09, 54.55)	0.43 (0.484)	18.44 (13.83, 23.05)	13.57 (9.05, 18.1)	26.38 (0.071)
	**Etramp**	14.88 (9.52, 20.24)	12.21 (8.4, 16.03)	17.92 (0.216)	43.14 (29.41, 56.86)	32.14 (19.64, 44.64)	25.49 (0.126)	21.68 (16.78, 26.57)	9.87 (6.28, 13.9)	54.49 (0.0002f)
	**ACS5**	31.33 (24.1, 38.55)	20.16 (15.32, 25.0)	35.64 (0.006)	56.86 (43.14, 70.59)	60.0 (47.27, 72.73)	-5.52 (0.620)	24.24 (19.32, 29.55)	18.52 (13.43, 23.61)	23.61 (0.057)
	**EBA175**	35.12 (27.98, 42.26)	26.05 (20.69, 31.42)	25.81 (0.025)	52.94 (39.22, 66.67)	56.36 (43.64, 69.09)	-6.46 (0.624)	27.37 (22.46, 32.63)	22.42 (17.04, 27.8)	18.08 (0.097)
	**PfTramp**	26.19 (19.64, 33.33)	12.6 (8.78, 16.79)	51.91 (0.001)	47.06 (33.33, 60.78)	35.71 (23.21, 48.21)	24.11 (0.118)	18.18 (13.64, 22.73)	15.7 (11.21, 20.63)	13.68 (0.233)
	**GEXP18**	12.5 (7.74, 17.86)	7.28 (4.21, 10.34)	41.76 (0.035)	30.77 (19.23, 42.31)	18.18 (9.09, 29.09)	40.91 (0.067)	10.8 (7.32, 14.63)	4.5 (1.8, 7.21)	58.3 (0.003)
	**PfRH2**	16.17 (10.78, 22.16)	8.98 (5.47, 12.5)	44.43 (0.014)	29.41 (17.65, 41.18)	29.09 (18.18, 41.82)	1.09 (0.471)	16.85 (12.54, 21.15)	9.09 (5.45, 13.18)	46.03 (0.004)
	**PfRH5**	19.39 (13.33, 25.45)	12.94 (9.02, 17.25)	33.27 (0.043)	30.0 (18.0, 42.0)	37.04 (24.07, 50.0)	-23.46 (0.765)	16.61 (12.37, 21.2)	11.01 (6.88, 15.14)	33.71 (0.035)

	**P1**	11.31 (6.55, 16.07)	6.49 (3.82, 9.54)	42.63 (0.047)	21.15 (9.62, 32.69)	17.86 (8.93, 28.57)	15.58 (0.355)	13.54 (9.72, 17.71)	5.83 (3.14, 8.97)	56.95 (0.001)
	**P39**	6.67 (3.03, 10.91)	9.34 (5.84, 12.84)	-40.08 (0.845)	17.31 (7.69, 28.85)	10.91 (3.64, 20.0)	36.97 (0.162)	8.04 (4.9, 11.19)	7.76 (4.57, 11.42)	3.47 (0.448)
	**P8**	8.93 (4.76, 13.69)	5.38 (2.69, 8.46)	39.69 (0.085)	11.54 (3.85, 21.15)	14.29 (5.36, 23.21)	-23.81 (0.667)	6.29 (3.5, 9.44)	7.69 (4.52, 11.31)	-22.22 (0.722)
	**PD**	13.17 (8.38, 18.56)	10.81 (7.34, 14.67)	17.94 (0.233)	19.61 (9.8, 31.37)	16.36 (7.27, 27.27)	16.55 (0.349)	13.78 (9.89, 18.02)	10.0 (6.36, 14.09)	27.44 (0.093)
	**DBL3-4**	13.25 (8.43, 18.67)	5.95 (3.17, 9.13)	55.09 (0.007)	36.0 (24.0, 50.0)	24.07 (12.96, 35.19)	33.13 (0.086)	13.82 (9.82, 18.18)	8.26 (5.05, 11.93)	40.25 (0.024)
	**All peptides**	21.18 (15.29, 27.65)	18.56 (14.02, 23.48)	12.35 (0.249)	33.33 (20.37, 46.3)	28.57 (17.86, 41.07)	14.29 (0.298)	23.88 (19.03, 29.07)	17.62 (12.78, 22.91)	26.2 (0.039)

Supplementary Table 10. Comparison of temporal patterns in clinical cases from health facilities and seroprevalence in pregnant women at first antenatal care visit.

Pearson CC statistics and time lag of the comparison between the temporal changes in seropositivity of different antibodies for different parity selections of pregnant women at first ANC visit and in clinical cases from passive surveillance.

	All prenatal		Primigravid women		Multigravid women		HIV+		HIV-		Primigravid HIV-	
**Antigen**	**Pearson CC**	**Time lag days**	**Pearson CC**	**Time lag days**	**Pearson CC**	**Time lag days**	**Pearson CC**	**Time lag days**	**Pearson CC**	**Time lag days**	**Pearson CC**	**Time lag days**
**MSP1**	0.55 (0.21, 0.64)	195	0.35 (-0.02, 0.50)	135	0.58 (0.18, 0.65)	282	0.56 (0.07, 0.62)	219	0.49 (0.14, 0.58)	132	0.30 (-0.07, 0.47)	129
**HSP40**	0.55 (0.08, 0.64)	282	0.52 (0.09, 0.61)	105	0.51 (0.10, 0.59)	282	0.26 (-0.06, 0.46)	273	0.58 (0.07, 0.66)	198	0.54 (0.16, 0.61)	129
**Etramp**	0.47 (0.01, 0.58)	282	0.37 (-0.01, 0.57)	96	0.32 (-0.01, 0.49)	282	0.56 (0.17, 0.66)	267	0.36 (0.03, 0.52)	132	0.39 (-0.08, 0.54)	279
**ACS5**	0.67 (0.31, 0.72)	144	0.55 (0.01, 0.65)	195	0.62 (0.17, 0.67)	144	0.65 (0.16, 0.66)	180	0.67 (0.20, 0.69)	144	0.33 (-0.07, 0.51)	126
**EBA175**	0.68 (0.26, 0.70)	186	0.35 (0.07, 0.52)	135	0.55 (0.10, 0.62)	282	0.67 (0.17, 0.71)	267	0.59 (0.18, 0.63)	132	0.34 (-0.07, 0.49)	135
**PfTramp**	0.41 (0.07, 0.55)	282	0.53 (0.08, 0.64)	135	0.39 (0.11, 0.56)	282	0.26 (-0.20, 0.47)	282	0.47 (0.05, 0.56)	132	0.52 (0.13, 0.61)	135
**GEXP18**	0.53 (0.11, 0.63)	198	0.58 (0.08, 0.67)	195	0.39 (-0.03, 0.52)	198	0.23 (-0.14, 0.44)	192	0.56 (0.14, 0.66)	210	0.52 (0.04, 0.61)	96
**PfRH2**	0.60 (0.11, 0.68)	171	0.61 (0.14, 0.70)	279	0.54 (0.10, 0.65)	162	0.44 (-0.01, 0.56)	282	0.58 (0.12, 0.66)	171	0.47 (0.02, 0.62)	279
**PfRH5**	0.62 (0.18, 0.68)	132	0.51 (0.04, 0.63)	282	0.60 (0.20, 0.68)	132	0.69 (0.20, 0.73)	273	0.56 (0.17, 0.65)	132	0.41 (-0.03, 0.52)	282
**P1**	0.73 (0.23, 0.75)	213	0.56 (0.00, 0.67)	222	0.62 (0.17, 0.68)	210	0.39 (-0.04, 0.54)	276	0.76 (0.19, 0.76)	240	0.60 (-0.06, 0.68)	240
**P39**	0.23 (-0.05, 0.52)	282	0.00 (-0.24, 0.35)	282	0.37 (-0.01, 0.52)	165	0.50 (0.04, 0.62)	255	0.01 (-0.26, 0.33)	159	-0.08 (-0.25, 0.43)	15
**P8**	0.42 (0.00, 0.53)	129	0.22 (-0.17, 0.47)	36	0.38 (0.00, 0.52)	132	0.42 (-0.11, 0.52)	282	0.33 (-0.07, 0.49)	120	0.26 (-0.18, 0.49)	81
**PD**	0.54 (0.10, 0.64)	282	0.43 (-0.04, 0.59)	105	0.54 (0.12, 0.64)	282	0.67 (0.15, 0.72)	282	0.29 (-0.03, 0.47)	132	0.37 (-0.05, 0.55)	105
**DBL3-4**	0.75 (0.25, 0.78)	213	0.68 (0.13, 0.72)	276	0.69 (0.17, 0.72)	204	0.53 (0.09, 0.62)	273	0.74 (0.21, 0.78)	213	0.70 (0.12, 0.72)	276
**All VAR2CSA peptides**	0.50 (0.03, 0.58)	282	0.33 (-0.06, 0.50)	279	0.48 (0.03, 0.56)	162	0.56 (0.09, 0.65)	282	0.17 (-0.16, 0.46)	132	0.28 (-0.10, 0.49)	282

Supplementary Table 11. Consistency of spatial clustering between *Plasmodium falciparum* qPCR positivity rates in children 2-10 and anti-*Plasmodium falciparum* seroprevalence in pregnant women at first antenatal care visit.

$\chi 2$ statistics of the differences between the 2-point cross correlation functions of PCR infections in children and the serostatus of pregnant women and the 2-point auto correlation function PCR infections in children.

**Antigen**	**Year 1**	**Year 2**	**Year 3**
**MSP1**	0.56	1.07	0.82
**HSP40**	0.53	1.12	0.84
**Etramp**	0.59	0.94	0.60
**ACS5**	0.60	1.16	0.75
**EBA175**	0.57	1.09	0.60
**PfTramp**	0.73	1.28	0.72
**GEXP18**	0.64	0.80	0.57
**PfRH2**	0.56	1.20	0.61
**PfRH5**	0.53	0.90	0.63
**P1**	0.80	1.60	0.63
**P39**	0.67	0.91	0.65
**P8**	0.57	1.45	0.81
**PD**	0.54	1.17	0.50
**DBL3-4**	0.59	1.39	0.72
**All VAR2CSA peptides**	0.76	1.52	0.60

Supplementary Table 12. *Plasmodium falciparum* antigens included in the quantitative suspension array assay panel.

Antigen	Rationale	Location	Longevity	Antigen	Coupling concentration (µg/5000 beads)	Producer	Ref.
**MSP1.19**	General Pf antigen, cummulative exposure	Merozoite surface	Long-lasting	Recombinant	30	ICGEB, Virander Chauhan	3
**HSP40 (Ag1)**	General Pf antigen, recent exposure	Infected red blood cell /gametocyte	Short-lasting	Recombinant	30	LSHTM, Chris Drakeley, Kevin Tetteh	4
**ETRAMP5 (Ag1)**	General Pf antigen, recent exposure	Infected red blood cell/PVM	Short-lasting	Recombinant	30	LSHTM, Chris Drakeley, Kevin Tetteh	4
**ACS5 (Ag3)**	General Pf antigen, recent exposure	Infected red blood cell/PVM	Short-lasting	Recombinant	30	LSHTM, Chris Drakeley, Kevin Tetteh	4
**EBA175 (region II F2)**	General Pf antigen	Merozoite	Short-lasting	Recombinant	30	ICGEB, Chetan Chitnis	5
**PfTRAMP**	General Pf antigen	Merozoite	Short-lasting	Recombinant	30	ICGEB, Chetan Chitnis	6
**GEXP18**	General Pf antigen, recent exposure	Gametocyte	Short-lasting	Recombinant	58	LSHTM, Chris Drakeley, Kevin Tetteh	4
**RH2 (2030)**	General Pf antigen	Merozoite	Short-lasting	Recombinant	30	ICGEB, Deepak Gaur	7
**RH5**	General Pf antigen	Merozoite	Short-lasting	Recombinant	30	ICGEB, Deepak Gaur	8
**P1 (VAR2CSA NTS)**	Pregnancy-specific Pf antigen, boosted by infection, cummulative exposure	Infected red blood cell (placenta)	Long-lasting	Peptide	1700	GL BioChem	1
**P39 (VAR2CSA DBL5ε)**	Pregnancy-specific Pf antigen, boosted by infection, cummulative exposure	Infected red blood cell (placenta)	Long-lasting	Peptide	1700	GL BioChem	1
**P8 (VAR2CSA ID1)**	Pregnancy-specific Pf antigen, boosted by infection	Infected red blood cell (placenta)	Short-lasting	Peptide	1700	GL BioChem	1
**PD (VAR2CSA ID1)**	Pregnancy-specific Pf antigen, boosted by infection	Infected red blood cell (placenta)	Short-lasting	Peptide	1700	GL BioChem	1
**DBL3-4 (VAR2CSA DBL3x-DBL4ε)**	Pregnancy-specific Pf antigen, highly conserved, not vaccine candidate	Infected red blood cell (placenta)	Short-lasting	Recombinant	30	INSERM, Benoit Gamain	9,10

Pf = Plasmodium falciparum - PVM = parasitophorous vacuole membrane - LSHTM = London School of Hygiene and Tropical Medicine - ICGEB = International Centre for Genetic Engineering and Biotechnology - INSERM = Institut National de la Santé et de la Recherche Médicale

Supplementary Table 13. Characteristics of subpopulations of pregnant women at first antenatal care visit.

Characteristics of the different populations of pregnant women analysed in this study. Sample size: number of women in this population; Mean age: mean age of the population; LLIN usage: usage of long-lasting insecticide treated nets during this pregnancy; IRS: indoor residual spraying conducted in their households during this pregnancy; Mean gest. weeks: average number of gestation weeks at the first ANC visit; Mean parity: average number of pregnancies; HIV positivity: percentage of HIV-positive women; Geom. mean p. density: geometric mean of the parasite densities of the qPCR-positive women from the group; qPCR positivity: qPCR positivity rate; RDT positivity: percentage of women with densities>100 parasites/μL; Antimalaric: fraction of women recently treated with any antimalarial drug during this pregnancy; MDA: fraction of women that participated in a massive drug administration during this pregnancy; Hemog.: average hemoglobin levels; Temp.: average body temperature; Malaria prev. year: percentage of women that were infected with malaria in this pregnancy. Anemia: percentage of women with anemia.

	**All prenatal**	**Primigravidae**	**Multigravidae**	**HIV+**	**HIV-**	**Primigravidae HIV-**
**Sample size**	6471	1754	4717	1872	4599	1548
**Mean age**	25.02	18.73	27.36	28.4	23.65	18.48
**LLIN usage (%)**	74.13	98.92	68.57	98.8	61.96	90.07
**IRS (%)**	84.51	90.51	67.29	85.14	66.82	80.83
**Mean gest. weeks**	20.43	18.97	20.98	20.32	20.48	18.99
**Mean parity**	2.74	1	3.39	3.38	2.48	1
**HIV positivity (%)**	28.93	11.74	35.32	100	0	0
**Geom. mean p. density**	36.68	103.3	21.22	65.45	30.67	92.07
**qPCR positivity (%)**	7.46	9.52	6.7	6.09	8.02	9.63
**RDT positivity (%)**	3.28	6.04	2.25	2.99	3.39	6.07
**Antimalaric (%)**	5.39	5.51	5.16	1.88	6.42	5.74
**MDA (%)**	1.07	1.32	0.98	0.19	1.42	1.49
**Hemog.**	10.9	10.97	10.88	10.48	11.08	11.03
**Temp.**	36.45	36.46	36.45	36.47	36.45	36.46
**Malaria prev. year (%)**	0.44	0.31	0.49	0.62	0.37	0.35
**Anemia (%)**	49.05	47.03	49.8	60.41	44.41	45.1

References

1 Fonseca, A. M. *et al.* VAR2CSA Serology to Detect Plasmodium falciparum Transmission Patterns in Pregnancy. *Emerg Infect Dis* **25**, 1851-1860 (2019). https://doi.org:10.3201/eid2510.181177

2 WHO. WHO malaria terminology, 2021 update. (Geneva: World Health Organization, 2021).

3 Mazumdar, S., Sachdeva, S., Chauhan, V. S. & Yazdani, S. S. Identification of cultivation condition to produce correctly folded form of a malaria vaccine based on Plasmodium falciparum merozoite surface protein-1 in Escherichia coli. *Bioprocess Biosyst Eng* **33**, 719-730 (2010). https://doi.org:10.1007/s00449-009-0394-x

4 Helb, D. A. *et al.* Novel serologic biomarkers provide accurate estimates of recent Plasmodium falciparum exposure for individuals and communities. *Proc Natl Acad Sci U S A* **112**, E4438-4447 (2015). https://doi.org:10.1073/pnas.1501705112

5 Pandey, K. C. *et al.* Bacterially expressed and refolded receptor binding domain of Plasmodium falciparum EBA-175 elicits invasion inhibitory antibodies. *Mol Biochem Parasitol* **123**, 23-33 (2002). https://doi.org:10.1016/s0166-6851(02)00122-6

6 Siddiqui, F. A. *et al.* A thrombospondin structural repeat containing rhoptry protein from Plasmodium falciparum mediates erythrocyte invasion. *Cell Microbiol* **15**, 1341-1356 (2013). https://doi.org:10.1111/cmi.12118

7 Sahar, T. *et al.* Plasmodium falciparum reticulocyte binding-like homologue protein 2 (PfRH2) is a key adhesive molecule involved in erythrocyte invasion. *PLoS One* **6**, e17102 (2011). https://doi.org:10.1371/journal.pone.0017102

8 Reddy, K. S. *et al.* Bacterially expressed full-length recombinant Plasmodium falciparum RH5 protein binds erythrocytes and elicits potent strain-transcending parasite-neutralizing antibodies. *Infect Immun* **82**, 152-164 (2014). https://doi.org:10.1128/iai.00970-13

9 Chêne, A. *et al.* Down-selection of the VAR2CSA DBL1-2 expressed in E. coli as a lead antigen for placental malaria vaccine development. *NPJ Vaccines* **3**, 28 (2018). https://doi.org:10.1038/s41541-018-0064-6

10 Gangnard, S. *et al.* Structure of the DBL3X-DBL4ε region of the VAR2CSA placental malaria vaccine candidate: insight into DBL domain interactions. *Sci Rep* **5**, 14868 (2015). https://doi.org:10.1038/srep14868

11 WHO. WHO malaria terminology, 2021 update. (Geneva: World Health Organization, 2021).

12 Matamoros E., Majewski, M. & Pujol, A. *Epidemiological Foci Relating Infections by Distance (EpiFRIenDs) for R*, https://github.com/arnaupujol/repifriends

13 Pujol, A. *Epidemiological Foci Relating Infections by Distance (EpiFRIenDs)*, https://github.com/arnaupujol/epifriends

14 Knebe, A. *et al.* Haloes gone MAD★: The Halo-Finder Comparison Project. *Monthly Notices of the Royal Astronomical Society* **415**, 2293-2318 (2011).

15 Ester, M., Kriegel, H. P., Sander, J. & Xiaowei, X. (AAAI Press, Menlo Park, CA, United States).

16 SaTScan v7.0: Software for the spatial and space-time scan statistics (2006).

17 Kulldorff, M. A spatial scan statistic. *Communications in Statistics - Theory and Methods* **26**, 1481-1496 (1997).

18 Kulldorff, M. & Nagarwalla, N. Spatial disease clusters: detection and inference. *Stat Med* **14**, 799-810 (1995).
