# Supplementary material for: Detecting temporal and spatial malaria patterns from first antenatal care visits
Source: Nat Commun. 2023 Jul 6;14:4004. doi: 10.1038/s41467-023-39662-4 (PMC10326053; doi:10.1038/s41467-023-39662-4)
Supplement: Supplementary file 3 — Description of Additional Supplementary Files [file 41467_2023_39662_MOESM3_ESM.pdf]

Description of Additional Supplementary Files

File Name: Supplementary Movie 1
Description: Temporal variation of the spatial distribution of the households of pregnant women at first ANC visits using temporal windows of one month. Grey dots represent qPCR negative cases, blue dots represent qPCR positive cases and red dots represent qPCR positive cases that belong to a hotspot. Geographic maps were generated from the public OpenStreetMap data, available under the Open Database License

File Name: Supplementary Movie 2
Description: Temporal variation of the spatial distribution of the households of children under 5 years of age attending health facilities using temporal windows of one month. Grey dots represent RDT negative cases, blue dots represent RDT positive cases and red dots represent RDT positive cases that belong to a hotspot. Geographic maps were generated from the public OpenStreetMap data, available under the Open Database License

File Name: Supplementary Movie 3
Description: Temporal variation of the spatial distribution of the households of pregnant women at first ANC visits using temporal windows of one month. Grey dots represent DBL3-4 seronegative women, blue dots represent DBL3-4 seropositive cases, and red dots represent DBL3-4 seropositive cases that belong to a serological cluster. Geographic maps were generated from the public OpenStreetMap data, available under the Open Database License.
