# Supplementary figures and images for: Detecting temporal and spatial malaria patterns from first antenatal care visits
Source: Nat Commun. 2023 Jul 6;14:4004. doi: 10.1038/s41467-023-39662-4 (PMC10326053; doi:10.1038/s41467-023-39662-4)

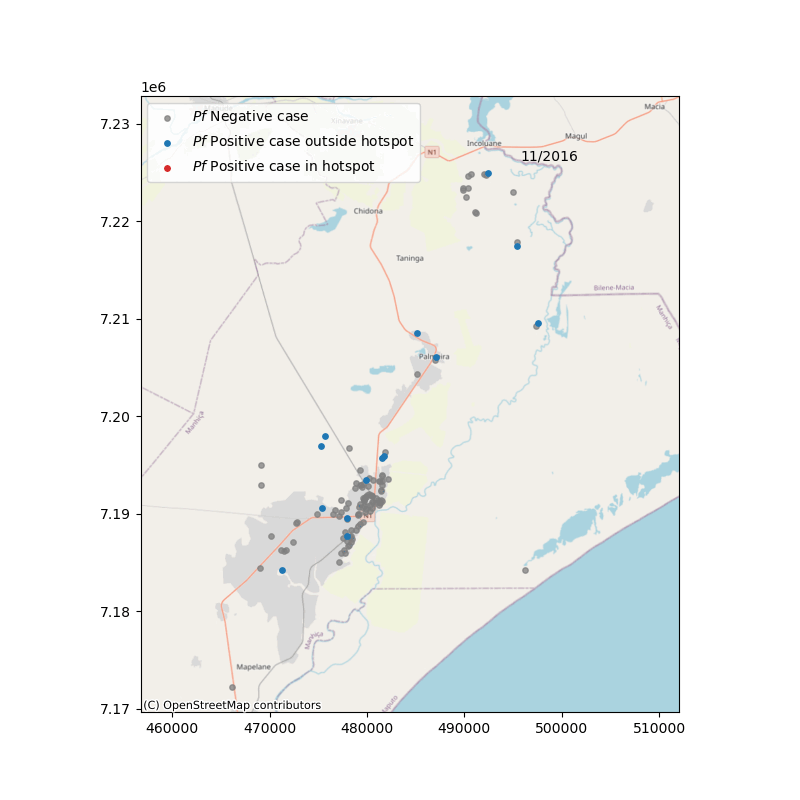

Supplement: Supplementary file 4 — Supplementary Movie 1 [file 41467_2023_39662_MOESM4_ESM.gif]

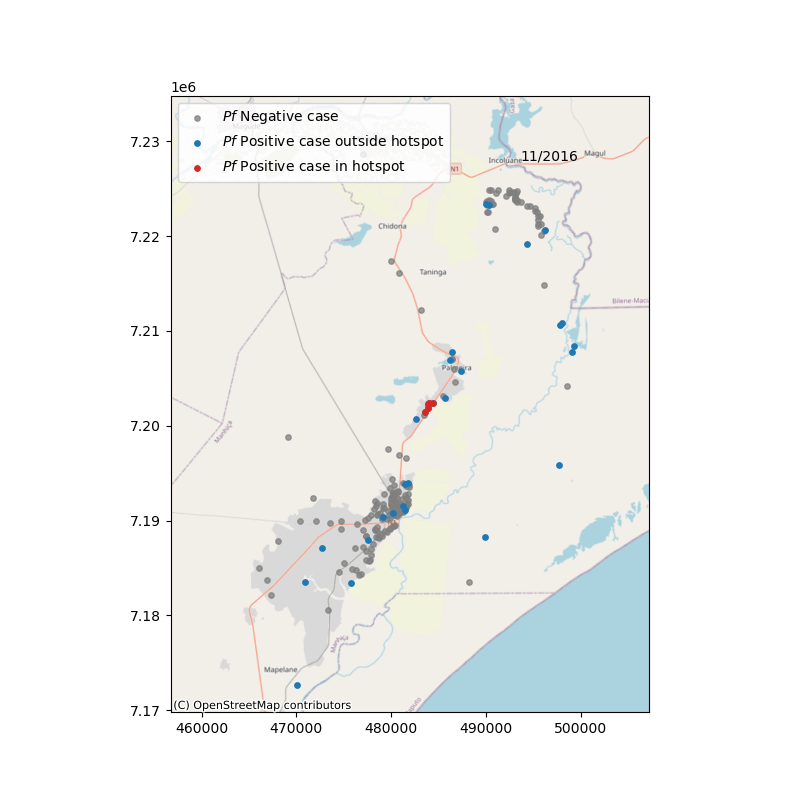

Supplement: Supplementary file 5 — Supplementary Movie 2 [file 41467_2023_39662_MOESM5_ESM.gif]

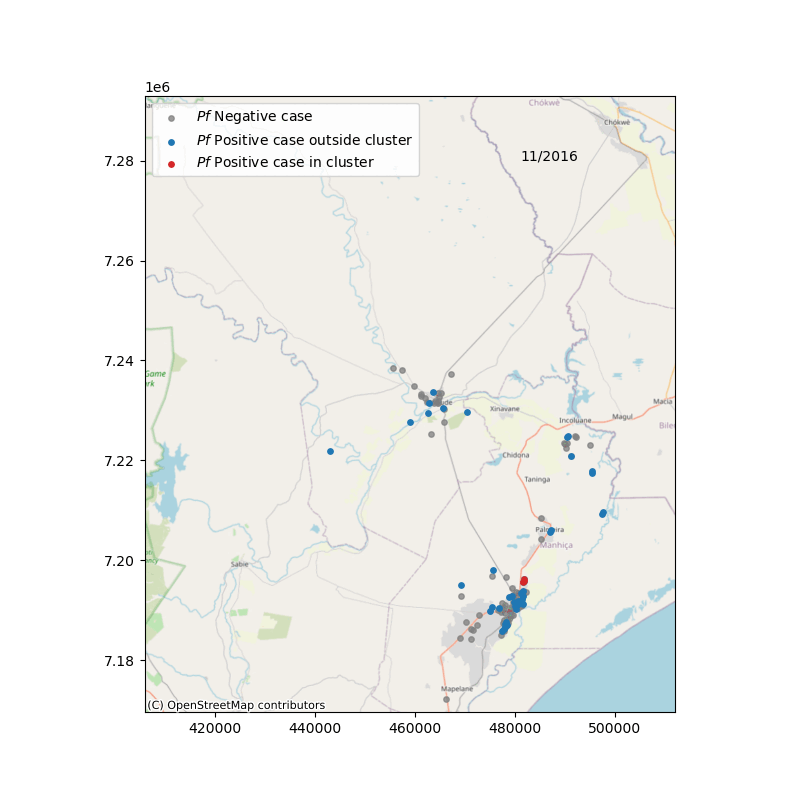

Supplement: Supplementary file 6 — Supplementary Movie 3 [file 41467_2023_39662_MOESM6_ESM.gif]
