# Supplementary material for: Detecting temporal and spatial malaria patterns from first antenatal care visits
Source: Nat Commun. 2023 Jul 6;14:4004. doi: 10.1038/s41467-023-39662-4 (PMC10326053; doi:10.1038/s41467-023-39662-4)
Supplement: Supplementary file 7 — Reporting Summary [file 41467_2023_39662_MOESM7_ESM.pdf]

nature portfolio

Corresponding author(s): Alfredo Mayor

Last updated by author(s): Jun 14, 2023

Reporting Summary

Nature Portfolio wishes to improve the reproducibility of the work that we publish. This form provides structure for consistency and transparency in reporting. For further information on Nature Portfolio policies, see our Editorial Policies and the Editorial Policy Checklist.

Statistics

For all statistical analyses, confirm that the following items are present in the figure legend, table legend, main text, or Methods section.

n/a	Confirmed	
☐	☒	The exact sample size (*n*) for each experimental group/condition, given as a discrete number and unit of measurement
☐	☒	A statement on whether measurements were taken from distinct samples or whether the same sample was measured repeatedly
☐	☒	The statistical test(s) used AND whether they are one- or two-sided<br>*Only common tests should be described solely by name; describe more complex techniques in the Methods section.*
☐	☒	A description of all covariates tested
☐	☒	A description of any assumptions or corrections, such as tests of normality and adjustment for multiple comparisons
☐	☒	A full description of the statistical parameters including central tendency (e.g. means) or other basic estimates (e.g. regression coefficient) AND variation (e.g. standard deviation) or associated estimates of uncertainty (e.g. confidence intervals)
☐	☒	For null hypothesis testing, the test statistic (e.g. *F*, *t*, *r*) with confidence intervals, effect sizes, degrees of freedom and *P* value noted<br>*Give P values as exact values whenever suitable.*
☐	☒	For Bayesian analysis, information on the choice of priors and Markov chain Monte Carlo settings
☐	☒	For hierarchical and complex designs, identification of the appropriate level for tests and full reporting of outcomes
☐	☒	Estimates of effect sizes (e.g. Cohen's *d*, Pearson's *r*), indicating how they were calculated

Our web collection on statistics for biologists contains articles on many of the points above.

Software and code

Policy information about availability of computer code

Data collection	The national District Health Information System 2 (DHIS2) and the Paediatric Outpatient Morbidity Surveillance System (OPMSS) platforms were used for the data collection and registration.
Data analysis	The open source code EpiFRIenDs v1.0 and SaTScan 10.1 was used for the hotspot analysis. Custom codes were used for the whole data analysis using Python 3.8.12, Jupyter Lab 3.1.14, R 4.2.1. Custom code used was made public in repository anc_surveillance tools v1.0 (https://github.com/arnaupujol/anc_surveillance_tools), that requires and points to other repositories made public (stat_tools v1.0, pregmal_pytools v1.0, spatial_tools v1.0 and genomics v1.0 from the GitHub page https://github.com/arnaupujol)

For manuscripts utilizing custom algorithms or software that are central to the research but not yet described in published literature, software must be made available to editors and reviewers. We strongly encourage code deposition in a community repository (e.g. GitHub). See the Nature Portfolio guidelines for submitting code & software for further information.

Data

Policy information about availability of data

All manuscripts must include a data availability statement. This statement should provide the following information, where applicable:

- Accession codes, unique identifiers, or web links for publicly available datasets
- A description of any restrictions on data availability
- For clinical datasets or third party data, please ensure that the statement adheres to our policy

Data was collected and registered by the District Health Information System 2 (DHIS2) and the Paediatric Outpatient Morbidity Surveillance System (OPMSS) from the national health system in Mozambique.
The raw epidemiological, clinical and GPS data are protected and are not available due to personal data privacy laws. The datasets generated and/or analysed during the current study are available under restricted access for data privacy laws involving personal data. Access can be obtained for research purposes by requesting to the corresponding author by email, specifying contact details, affiliation and purpose of the request. All requests for processed data used in this study are reviewed in a three-month timeframe by Manhiça Health Research Center to verify if the request is subject to any intellectual property or confidentiality obligations. Patient-related data not included in the paper might be subject to patient confidentiality. Any data that can be shared will be released via a Data Transfer Agreement.

Human research participants

Policy information about studies involving human research participants and Sex and Gender in Research.

Reporting on sex and gender

The study included data from pregnant women at antenatal care visits and children from cross-sectional surveys and health facilities. The aim of the study was to compare statistics between pregnant women and children in order to assess the potential of pregnant women at first antenatal care visit for malaria surveillance. In this sense, the biological condition used to select this population (regardless of sex or gender consideration) was the pregnancy status. Children from cross-sectional surveys were selected regardless of sex or gender (with a fraction of 49.2% of male and 50.3% of female self-reported or reported by their adult representatives, with 0.5% of unavailable information) to obtain a sub-representation of the census. All symptomatic children from health facilities were considered to account for all passively detected malaria cases and information of sex or gender was not collected since it was personal data that was not relevant for the analysis.

Population characteristics

The main factors of the analysis taken into account were based a previous study from the same samples, showing that the main factors affecting qPCR positivity rates were HIV status and gravidity (Matambisso G. et al. Gravidity and malaria trends interact to modify P. falciparum densities and detectability in pregnancy: a 3-year prospective multisite observational study. BMC Med 20, 396 (2022). https://doi.org:10.1186/s12916-022-02597-6).
Descriptions of covariate-relevant population characteristics are shown in Supplementary Table 13. Mean age was correlated with gravidity (18.73 for primigravidae, 27.36 for multigravidae) due to the fact that gravidity increases with age for all participants. Primigravid and HIV+ women reported higher LLIN usage (98.92% and 98.8%) than HIV- and multigravidae (68.57% and 61.96%), and similar trends were found for IRS coverage (90.51% and 85.14% versus 67.29% and 66.82%), but the high LLIN usage and IRS coverage in primigravid women do not allow a statistically significant stratification of these factors. Primigravid women showed lower HIV positivity than multigravid women (11.74% versus 35.32%), and primigravid HIV- were studied in order to study the potential effect of such correlations. Geometric mean of parasite densities were higher among primigravid (103.3) and HIV+ (65.45) positive women than among multigravid (21.22) and HIV- (30.67) women, probably due to their lower levels of immunity.

Recruitment

Data was obtained from 6,471 pregnant women (Supplementary figure S1), residing in the study area, who attended their first ANC visit at Manhiça District Hospital, Ilha Josina Health Centre, or Magude Health Centre. Pregnant women were only included in the study if they gave consent to participate in the study and if they met the inclusion criteria: attending a first routine antenatal care (ANC) visit or a visit for delivery, and being from the study area with a permanent identification number issued by the demographic surveillance system or residing in the area. All the pregnant women at ANC who agreed to participate were requested to donate finger prick blood onto filter paper for detection of parasite DNA and antibodies by quantitative polymerase chain reaction (qPCR) and quantitative suspension array based on Luminex technology, respectively, together with routine tests. A brief form was filled in with information including the visit date, age, gravidity, area of residence and other information such as the use of preventive tools.
Weekly numbers of RDT-positive clinical malaria cases among children <5 years old attending the three health facilities (n=15,467) were obtained from the District Health Information System 2 (DHIS2). In Manhiça district, 37,131 RDT and microscopy results from children <5 years attending health facilities were available from the paediatric outpatient morbidity surveillance system (OPMSS). Data from 9,362 children aged 2-10 years was collected in age-stratified cross-sectional surveys conducted every May from 2015 to 2019. Geo-localization of pregnant women and children was obtained from a local health and demographic surveillance system using their permanent or family identification number, from their household identification number or by registering the geo-localization of the households.
For the cross-sectional surveys, a random age-stratified sample of the population of Manhiça (2,373 km2, 180,000 individuals) and Magude (6,961 km2, 60,000 individuals) districts, with oversampling of children under 15 years old, was selected from the Centro de Investigação em Saúde de Manhiça (CISM) Demographic Health Surveillance System (DHSS) census. A standardised electronic questionnaire using REDCap was completed for each participant with basic socio-demographic, clinical and vector control information. From all consenting participants finger-prick blood samples were collected for malaria diagnostics by microscopy, rapid diagnostic test (RDT) and qPCR. The collection was conducted around every May (approximately between mid April and mid June) 2015-2019, but qPCR data from Manhiça from 2017 was not available for this study.
Geographic information system (GIS) locations from participants were collected from the national census database using their permanent or family identification number (information that is updated twice per year), or in absence of this data, from

their household identification number or by registering the GIS location of the households in place in the case of the national cross-sectional surveys.
Potential biases could come from participant consents given in a non-representative manner in both pregnant women at antenatal care visits or children in cross-sectional surveys. However, a weight was applied to all members of the cross-sectional surveys to account for their representativity with respect to the census. Women not attending ANC tend to be older, live in rural settings, and be of lower socio-economic status than attending women, which are all risk factors for malaria. However, this selection bias is likely to be low in sub-Saharan Africa due to high ANC attendance, and in any case the goal of the study is to assess the potential of malaria surveillance using data from antenatal care visits, so these biases should be included in the analysis since they are expected to be present in the potential surveillance system.

Ethics oversight

All study protocols were approved by the institutional ethics committees at CISM and Barcelona Hospital Clínic, and the Mozambican Ministry of Health National Bioethics Committee. All data and biological samples were collected only if research participants (or representatives in the case of minors) gave full written informed consent.

Note that full information on the approval of the study protocol must also be provided in the manuscript.

Field-specific reporting

Please select the one below that is the best fit for your research. If you are not sure, read the appropriate sections before making your selection.

☒ Life sciences ☐ Behavioural & social sciences ☐ Ecological, evolutionary & environmental sciences

For a reference copy of the document with all sections, see nature.com/documents/nr-reporting-summary-flat.pdf

Life sciences study design

All studies must disclose on these points even when the disclosure is negative.

Sample size

Sample size of cross-sectional surveys from Manhiça district were estimated to detect a prevalence of parasitemia of 50% (conservative estimate with maximum imprecision) with a precision of ±10% approximately and a confidence coefficient of 95% per age group, from which we used data from children 2-10 years old that involved 5 age groups.
Sample size of cross-sectional surveys from Magude district were calculated based on the two sampling scenarios:
a) Simple random sampling: a sample of 384 participants per age group (980 in total) were
needed to detect a malaria prevalence of 50% (conservative estimate with maximum imprecision), with a precision of ±5% and a 95% level of confidence.
b) Multi-stage cluster random sampling: due to the logistical and resource constrains that would involve sampling a group large enough to detect the most conservative prevalence with maximum precision using multi-stage cluster random sampling, in this case, the sample size calculations were set to detect a prevalence of 20%, with a precision of ±10% and a 95% level of confidence, assuming a design effect (DEFF) of 2, a 10% non-response rate, and an average administrative post size of 50 people.

For the molecular detection of P. falciparum from pregnant women at antenatal care visits, all DBS were used from Ilha Josina (n=250/year) and a random selection of approximately 2700 samples per year in both Magude and Manhiça was used to allow for the estimation of the 95% confidence interval (95%CI) of annual P. falciparum positivity rates between 20 and 5% in each of the 3 sites, with a margin of error lower than or equal to the expected positivity rate.
Aggregated data from clinical cases included all cases, so no sample size estimation was required.

Data exclusions

Data exclusion criteria was pre-established before the study. Data was excluded if no consents from the participants were obtained. Antenatal care (ANC) visits for delivery were excluded due to the expected biases in burden levels due to intermittent preventive treatment administered after the first ANC visit. They were also excluded if they were not residing in the study area, since it could affect the spatial analysis of malaria burden.
Samples eluted for the multiplexed bead immunoassay were excluded if the colour of the filter paper remained red and the elution remained transparent. Antibody measurements were discarded if fewer than 20 counts per recombinant antigen and 10 per peptide were obtained. Percentage covariance between plates was calculated from a positive control (hyperimmune serum pool) and considered acceptable if below 30%.

Replication

The codes used to conduct the analysis will be made public to replicate the whole analysis. At least two authors of the study have conducted and validated the results on positivity rates and declines in malaria burden using the different data sources. All attempts at replication were successful. Results and performance of the open source Python code EpiFRIenDs were repeated and validated with another version of the code using R.

Randomization

Dried blood spot samples analysed in the multiplex bead based immunoassay were randomised by assigning a computer-generated random number to each sample and ordering them accordingly.
Different selections of pregnant women in HIV status and parity were used to account for the impact of such factors, which were identified as the most important factors of malaria burden in pregnant women in Matambisso et al. 2022, BMC Med, 20, 396. The analysis was done for different detectability thresholds to assess the impact of rapid diagnostic test (RDT) versus quantitative polymerase chain reaction (qPCR) results.

Blinding

Blinding was not relevant for our study because there was no clinical trial in the process. The analysis was conducted in the analysis of representative population that were equally trated. Tesing and sampling was conducted using the same protocol for all populations for the

exclusion criteria, selection, testing and treatment of participants. The goal of the study was to compare malaria trends in different sentinel groups following the common approaches for malaria surveillance.

Reporting for specific materials, systems and methods

We require information from authors about some types of materials, experimental systems and methods used in many studies. Here, indicate whether each material, system or method listed is relevant to your study. If you are not sure if a list item applies to your research, read the appropriate section before selecting a response.

Materials & experimental systems

n/a	Involved in the study	
☐	☒	Antibodies
☒	☐	Eukaryotic cell lines
☒	☐	Palaeontology and archaeology
☒	☐	Animals and other organisms
☐	☒	Clinical data
☐	☐	Dual use research of concern

Methods

n/a	Involved in the study	
☒	☐	ChIP-seq
☒	☐	Flow cytometry
☒	☐	MRI-based neuroimaging

Antibodies

Antibodies used

Immunoglobulin Gs (IgG) were detected and quantified in a multiplexed bead array using Luminex xMAP© technology (Luminex Corp., Austin TX), as described in Matambisso et al. 2022, BMC Med, 20, 396. In brief, magnetic beads were coupled to our panel including VAR2CSA antigens (Duffy binding-like recombinant domains DBL3-4, peptide P1 targeting the NTS region, peptides P8 and PD targeting ID1, and P39 targeting DBL5ε), general malaria antigens (19-kDa fragment of the merozoite surface protein-1 [MSP119], region II/F2 of erythrocyte-binding antigen-175 [EBA175], full-length P. falciparum reticulocyte binding-like homologue protein 2 and 5 [RH2 and RH5]), thrombospondin-related apical merozoite protein [PfTRAMP], and biomarkers of recent P. falciparum exposure (gametocyte exported protein 18 [GEXP18], acyl-CoA synthetase 5 [ACS5] ag3, early transcribed membrane protein 5 [ETRAMP5] ag1, and heat shock protein 40 [HSP40] ag1). Detailed information in Supplementary table 12. Primary IgGs were detected with Fc-specific anti-human IgG-biotin, produced in goat, polyclonal, supplied by Sigma-Aldrich, product number SAB3701279, and Streptavidin-R-Phycoerythrin (1 mg/ml), supplied by Sigma-Aldrich, product number 42250.

Validation

Optimal coupling concentration was determined for each antigen. Mean fluorescent intensity (MFI) for each antigen was measured in a 22-point 2-fold dilution series of a hyperimmune pool starting at a 1:10 dilution. The hyperimmune pool consisted of 60 sera from pregnant women from Mozambique selected for their high immunogenicity against the panel diluted in Luminex Buffer (Phosphate Buffered Saline [PBS], 1% BSA, 0.05% azide, pH 7.4). Coupled beads were quantified on a Guava PCA desktop cytometer (Guava, Hayward, CA) to confirm a high bead recovery (minimum 80%). A MFI of 25,000 or above at a 1:10 dilution and a standard curve fitting a 5PL regression model was considered a good coupling. Subsequently, coupled beads were tested in both singleplex and multiplex to reject potential cross-reactivity between antigens.
Six controls were included in each plate of the assay. To monitor the DBS punching and elution process, mock DBS made from plasma mixed with pooled serum from 45 malaria-immune Mozambican pregnant women. A standard curve was prepared from serum pooled from 35 malaria-immune Mozambican pregnant women in a 3-fold 14-point serial dilution starting at 1:100 with Luminex Buffer (Phosphate Buffered Saline [PBS], 1% BSA, 0.05% azide, pH 7.4). Three dilutions of the standard curve (1:500, 1:5,000 and 1:50,000) were also included to monitor variations between plates. National Institute for Biological Standards and Controls (NIBSC) serum against P. falciparum (First WHO Reference Reagent for Pf anti-malaria human serum, NIBSC 10/198) was included at 1:100 dilution in dH2O. Furthermore, negative control samples collected from malaria-naïve individuals and blank wells (Luminex Buffer) were included in all plates. To measure the unspecific binding of antibodies in the sample to BSA used to block the beads, beads coupled to BSA were included in the bead multiplex.

List of antigens used and their producers:
MSP1.19: produced by ICGEB (Virander Chauhan) [3]
HSP40 (Ag1): produced by LSHTM (Chris Drakeley, Kevin Tetteh) [4]
ETRAMP5 (Ag1): produced by LSHTM (Chris Drakeley, Kevin Tetteh) [4]
ACS5 (Ag3): produced by LSHTM (Chris Drakeley, Kevin Tetteh) [4]
EBA175 (region II F2): produced by ICGEB (Chetan Chitnis) [5]
PfTRAMP: produced by ICGEB (Chetan Chitnis) [6]
GEXP18: produced by LSHTM (Chris Drakeley, Kevin Tetteh) [4]
RH2 (2030): produced by ICGEB (Deepak Gaur) [7]
RH5: produced by ICGEB (Deepak Gaur) [8]
P1 (VAR2CSA NTS): produced by GL BioChem [2]
P39 (VAR2CSA DBL5ε): produced by GL BioChem [2]
P8 (VAR2CSA ID1): produced by GL BioChem [2]
PD (VAR2CSA ID1): produced by GL BioChem [2]
DBL3-4 (VAR2CSA DBL3x-DBL4ε): produced by INSERIM (Benoit Gamain) [9,10]

More detailed information can be found in Supplementary table 12.

References
[1] Mayor, A. et al. How hidden can malaria be in pregnant women? Diagnosis by microscopy, placental histology, polymerase chain

reaction and detection of histidine-rich protein 2 in plasma. Clin Infect Dis 54, 1561-1568 (2012). https://doi.org:10.1093/cid/cis236
[2] Fonseca, A. M. et al. VAR2CSA Serology to Detect Plasmodium falciparum Transmission Patterns in Pregnancy. Emerg Infect Dis 25, 1851-1860 (2019). https://doi.org:10.3201/eid2510.181177
[3] Mazumdar, S., Sachdeva, S., Chauhan, V. S. & Yazdani, S. S. Identification of cultivation condition to produce correctly folded form of a malaria vaccine based on Plasmodium falciparum merozoite surface protein-1 in Escherichia coli. Bioprocess Biosyst Eng 33, 719-730 (2010). https://doi.org:10.1007/s00449-009-0394-x
[4] Helb, D. A. et al. Novel serologic biomarkers provide accurate estimates of recent Plasmodium falciparum exposure for individuals and communities. Proc Natl Acad Sci U S A 112, E4438-4447 (2015). https://doi.org:10.1073/pnas.1501705112
[5] Pandey, K. C. et al. Bacterially expressed and refolded receptor binding domain of Plasmodium falciparum EBA-175 elicits invasion inhibitory antibodies. Mol Biochem Parasitol 123, 23-33 (2002). https://doi.org:10.1016/s0166-6851(02)00122-6
[6] Siddiqui, F. A. et al. A thrombospondin structural repeat containing rhoptry protein from Plasmodium falciparum mediates erythrocyte invasion. Cell Microbiol 15, 1341-1356 (2013). https://doi.org:10.1111/cmi.12118
[7] Sahar, T. et al. Plasmodium falciparum reticulocyte binding-like homologue protein 2 (PfRH2) is a key adhesive molecule involved in erythrocyte invasion. PLoS One 6, e17102 (2011). https://doi.org:10.1371/journal.pone.0017102
[8] Reddy, K. S. et al. Bacterially expressed full-length recombinant Plasmodium falciparum RH5 protein binds erythrocytes and elicits potent strain-transcending parasite-neutralizing antibodies. Infect Immun 82, 152-164 (2014). https://doi.org:10.1128/iai.00970-13
[9] Chêne, A. et al. Down-selection of the VAR2CSA DBL1-2 expressed in E. coli as a lead antigen for placental malaria vaccine development. NPJ Vaccines 3, 28 (2018). https://doi.org:10.1038/s41541-018-0064-6
[10] Gangnard, S. et al. Structure of the DBL3X-DBL4ε region of the VAR2CSA placental malaria vaccine candidate: insight into DBL domain interactions. Sci Rep 5, 14868 (2015). https://doi.org:10.1038/srep14868

Clinical data

Policy information about clinical studies

All manuscripts should comply with the ICMJE guidelines for publication of clinical research and a completed CONSORT checklist must be included with all submissions.

Clinical trial registration	Not applicable, there was no clinical trial.
Study protocol	Not applicable, there was no clinical trial.
Data collection	Weekly numbers of RDT-positive clinical malaria cases among children <5 years old attending the three health facilities (n=15,467) were obtained from the District Health Information System 2 (DHIS2). In Manhiça district, 37,131 RDT and microscopy results from children <5 years attending health facilities were available from the paediatric outpatient morbidity surveillance system (OPMSS). Data collected between 2016 and 2020 was included in the analysis. Geographic information system (GIS) locations from participants were collected from the national census database using their permanent or family identification number (information that is updated twice per year), or in absence of this data, from their household identification number.
Outcomes	The main outcome from clinical data was the weekly number of P. falciparum RDT or microscopy positive cases at the three health facilities, for each week from 2016 to 2020. The RDT and microscopy results from OPMSS was used for the hotspot analysis using the GPS location of the participants.

Dual use research of concern

Policy information about dual use research of concern

Hazards

Could the accidental, deliberate or reckless misuse of agents or technologies generated in the work, or the application of information presented in the manuscript, pose a threat to:

No	Yes	
☒	☐	Public health
☒	☐	National security
☒	☐	Crops and/or livestock
☒	☐	Ecosystems
☒	☐	Any other significant area

Experiments of concern

Does the work involve any of these experiments of concern:

No	Yes	
☒	☐	Demonstrate how to render a vaccine ineffective
☒	☐	Confer resistance to therapeutically useful antibiotics or antiviral agents
☒	☐	Enhance the virulence of a pathogen or render a nonpathogen virulent
☒	☐	Increase transmissibility of a pathogen
☒	☐	Alter the host range of a pathogen
☒	☐	Enable evasion of diagnostic/detection modalities
☒	☐	Enable the weaponization of a biological agent or toxin
☒	☐	Any other potentially harmful combination of experiments and agents
